# Thought disorder measured as random speech structure classifies negative symptoms and schizophrenia diagnosis 6 months in advance

**DOI:** 10.1038/s41537-017-0019-3

**Published:** 2017-04-13

**Authors:** Natália B. Mota, Mauro Copelli, Sidarta Ribeiro

**Affiliations:** 1 Brain Institute, Federal University of Rio Grande do Norte, UFRN, Natal, RN Brazil; 2 Physics Department, Federal University of Pernambuco, UFPE, Recife, PE Brazil

## Abstract

In chronic psychotic patients, word graph analysis shows potential as complementary psychiatric assessment. This analysis relies mostly on connectedness, a structural feature of speech that is anti-correlated with negative symptoms. Here we aimed to verify whether speech disorganization during the first clinical contact, as measured by graph connectedness, can correctly classify negative symptoms and the schizophrenia diagnosis 6 months in advance. Positive and negative syndrome scale scores and memory reports were collected from 21 patients undergoing first clinical contact for recent-onset psychosis, followed for 6 months to establish diagnosis, and compared to 21 well-matched healthy subjects. Each report was represented as a word-trajectory graph. Connectedness was measured by number of edges, number of nodes in the largest connected component and number of nodes in the largest strongly connected component. Similarities to random graphs were estimated. All connectedness attributes were combined into a single Disorganization Index weighted by the correlation with the positive and negative syndrome scale negative subscale, and used for classifications. Random-like connectedness was more prevalent among schizophrenia patients (64 × 5% in Control group, *p* = 0.0002). Connectedness from two kinds of memory reports (dream and negative image) explained 88% of negative symptoms variance (*p* < 0.0001). The Disorganization Index classified low vs. high severity of negative symptoms with 100% accuracy (area under the receiver operating characteristic curve = 1), and schizophrenia diagnosis with 91.67% accuracy (area under the receiver operating characteristic curve = 0.85). The index was validated in an independent cohort of chronic psychotic patients and controls (*N* = 60) (85% accuracy). Thus, speech disorganization during the first clinical contact correlates tightly with negative symptoms, and is quite discriminative of the schizophrenia diagnosis.

## Diagnosis: Early signs of speech problems indicative of thought disorder

Abnormal speech in someone showing early signs of psychosis can help doctors diagnose schizophrenia and its ‘negative’ symptoms. Natália Mota from the Federal University of Rio Grande do Norte, Brazil, and colleagues asked 21 people undergoing first clinical contact for recent-onset psychosis and 21 healthy controls to recall a dream or recent memory. They then analyzed the structure of the participants’ verbal reports using a mathematical technique. The patients were followed up during 6 months to establish a more formal diagnosis. The researchers found that those later diagnosed with schizophrenia exhibited more disorganized speech (almost random in structure) at the initial doctor’s visit than those later diagnosed with bipolar disorder. Less connected speech among people with schizophrenia was also indicative of more severe negative symptoms such as blunted emotions and lack of motivation.

## Introduction

Schizophrenia is associated with negative symptoms, major impacts on social behavior and poor prognosis.^1^ In particular, elevated negative symptoms are associated with low rates of recovery.^1, 2^ Formal thought disorder—which comprises poverty of speech, derailment, and incoherence—constitutes an important set of psychotic symptoms, and negative formal thought disorder is associated with the schizophrenia diagnosis even during first episode psychosis.^2, 3^ The early stages of the disease constitute a critical opportunity for prevention of major cognitive damage.^4^

Improved behavioral measures subjected to novel mathematical analyses are emerging as part of a new field that uses computational tools to better characterize psychiatric phenomena.^5–13^ A particularly useful example of such computational phenotyping is the assessment of verbal reports by graph analysis, which provides a precise and automated quantification of speech features that are related with negative symptoms^9^ and show potential to help the differential diagnosis of psychosis.^9, 10^ By representing each word as a node and the temporal sequence of consecutive words as directed edges, it is possible to calculate attributes that characterize graph structure.^9, 10^ The assessment of dream reports from chronic psychotic patients has shown that patients diagnosed with schizophrenia typically talk with fewer words than those diagnosed with bipolar disorder or matched controls.^9, 10^ Even when verbosity differences are controlled, negative symptoms are anti-correlated with various measures of word connectedness (such as number of edges, and the amount of nodes in the largest connected component—LCC and in the largest strongly connected component—LSC). Overall, the higher the graph connectedness, the lesser the negative symptoms.^9^

An interesting point is that dream reports were especially informative regarding the schizophrenia diagnosis and correlations with negative symptoms compared to reports from waking activities. The same graph attributes, when calculated from short-term memory reports produced by healthy children, were positively correlated with Intelligence Quotient and Theory of Mind scores, and could predict academic performance independently of other cognitive measures.^14^ Interestingly, reports related to long-term memories were not correlated with cognitive measurements.^14^ Altogether, these data add to the notion that word connectedness rises during healthy development, but not during the course of schizophrenia.^9, 10, 14^ Although this hypothesis can only be directly addressed with a longitudinal design, we found a positive exponentially saturating relationship between educational level and connectedness in healthy controls, in a cross-sectional study of a larger sample with a wide span of educational levels.^15^ Importantly, this education-dependent dynamics was blurred in the psychosis group.

The results led us to hypothesize that early markers of speech disorganization during recent-onset psychosis, such as decreased connectedness, may be able to correctly classify the severity of negative symptoms as well as the schizophrenia diagnosis. Here we tested four specific hypotheses: (1) Speech connectedness from dream reports^9^ and short-term memory reports^14^ can discriminate the schizophrenia diagnosis; (2) Patients in the schizophrenia group produce verbal reports less connected and more similar to random connectedness than those from Bipolar or Control groups; (3) Connectedness attributes correlate negatively with negative symptoms^9^; (4) A single index combining connectedness attributes highly correlated with negative symptoms will improve the schizophrenia diagnosis and the classification of negative symptom severity.

## Results

**Fig. 1:**
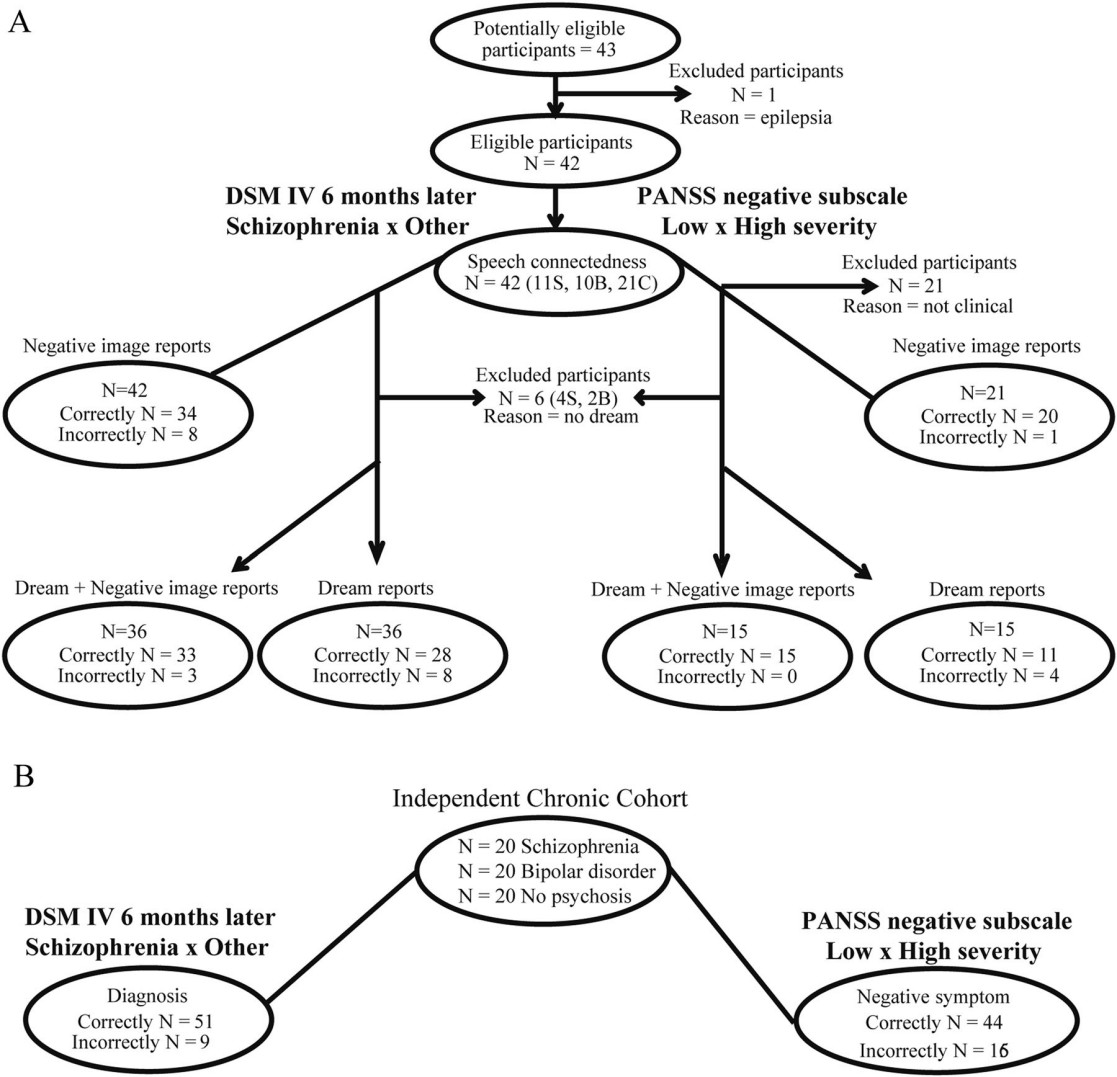
Illustrative diagrams of the flow of participants. **a** Using Dream + Negative image reports or only Negative image reports, or only Dream reports. Control subjects were excluded from positive and negative syndrome scale (PANSS) analyses because they were “not clinical”, i.e., they were not at clinical settings. **c** Through the validation in an independent cohort of chronic psychotic patients. Schizophrenia (S), Bipolar **b** and Control **c** groups

Patients seeking treatment for the first time for psychotic symptoms, without neurological or drug-related disorders, were interviewed in 2014 and 2015 (*N* = 21). After a 6 months follow-up, 11 patients were diagnosed with schizophrenia disorder, and 10 with Bipolar disorder (Table 1, Fig. 1a). The schizophrenia group used more atypical antipsychotic medications and less mood stabilizers than the Bipolar group (Table 1). As controls, healthy subjects matched for sex, age, and education were recruited and interviewed in public schools (*N* = 21). Despite the absence of significant differences regarding demographical characteristics (age, sex, educational level, and family income) or disease duration (Table 1), the schizophrenia group had substantially more males than the other groups, as well as a smaller educational level. For this reason our analyses included gender, years of education, age and chlorpromazine equivalent dose as potential confounding factors.

**Table 1:** Socio-economic and clinical information of Schizophrenia, Bipolar, and Control groups

Demographic Characteristics	Schizophrenia	Bipolar Disorder	Control	*p* Value S x (B + C)
Age (years)	14.64 ± 2.57	15.30 ± 3.77	15.43 ± 3.55	0.5837
Family Income (US$ per month)	326.14 ± 190.58	297.50 ± 166.94	368.42 ± 151.76	0.3746
Sex				
Male	82%	27%	45%	0.0542
Female	18%	73%	55%	
Years of Education (years)	5.73 ± 2.34	6.40 ± 3.77	8.05 ± 2.77	0.0810
Psychiatric Assessment	Schizophrenia	Bipolar Disorder		*p* Value: S x B
Medication				
Typical Antipsychotic	55%	60%		0.8008
Atypical Antipsychotic	82%	40%		0.0487
Mood Stabilizer	9%	70%		0.0041
Benzodiazepine	9%	10%		0.9435
Antidepressants	9%	20%		0.4755
Disease Duration (days)	339.36 ± 244.80	370.60 ± 306.08		1

Mean ± standard deviation of age in years, family income in USD per month, educational level in years, disease duration in days. Shown are the percentage of male and female subjects per group, and the percentage of subjects under specific types of medication. *P* values of Wilcoxon–Ranksum test or Chi-square test between Schizophrenia vs. Bipolar and Control groups (general information), or Schizophrenia vs. Bipolar group (clinical information). Group label according to diagnosis established after 6 months of follow-up

**Fig. 2:**
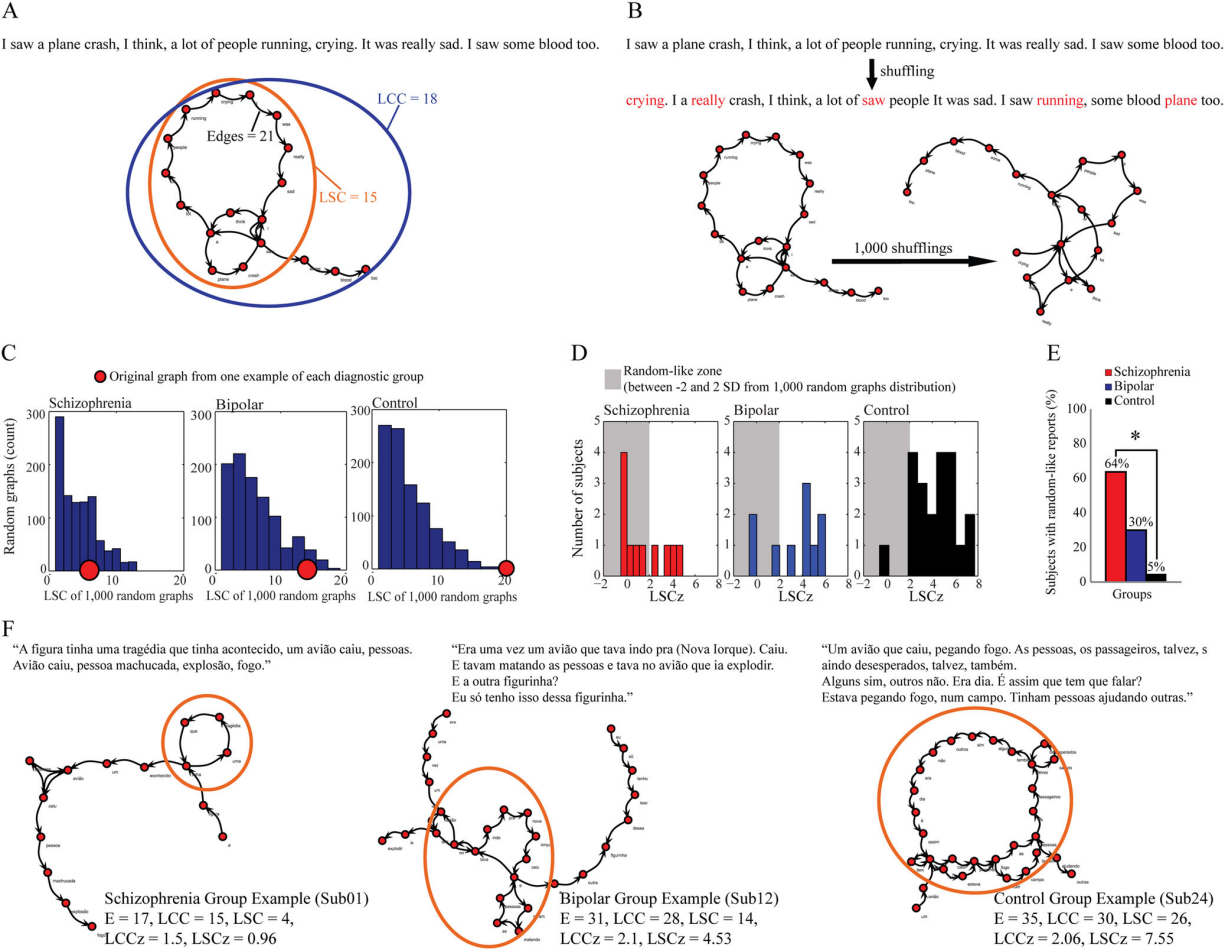
Speech graph connectedness attributes and random-like connectedness in schizophrenia. **a** Illustrative example of a text represented as a graph, showing connectedness attributes Edges, LCC, and LSC. **b** Illustrative example of random graphs created from an original report. By shuffling word order 1000 times, surrogated graphs maintained the same words but displayed a random word structure (displaced words in *red*). **c** Examples of one negative image report compared to 1000 random graphs for each group. Estimation of original LSC (*red dot*) distance from a 1000 random graph distribution (*blue* histogram) by *z*-score—LSCz. **d** LSCz histogram from each diagnostic group, considering as random-like speech those with LSCz = −2 until 2 (2 standard deviation from a random graph distribution). **e** Percentage of random-like reports in each diagnostic group (Asterisk means *p* < 0.05—*χ* ^2^ test). **f** Representative graphs for each group, obtained from negative image reports

Interviews included regular psychiatric anamnesis plus requests to report a dream, a memory of the day that preceded the dream, and the oldest memory recalled. Subjects were also requested to imagine and report a short story based on three affective images (one negative, one neutral, and one positive regarding affective valence).^14, 16^ All the reports were limited to 30 s by the interviewer. Whenever a subject interrupted a report before 30 s had elapsed, he/she was prompted by the interviewer to continue talking up to the time limit. The reports were audio recorded, transcribed and represented as graphs with each word represented as a node and the temporal sequence between words represented as directed edges (Fig. 2a).

Three connectedness attributes were calculated: Amount of edges (E); amount of nodes in the LCC, defined as the largest set of nodes directly or indirectly linked by some path; and the amount of nodes in the LSC, defined as the largest set of nodes directly or indirectly linked by reciprocal paths, so that all the nodes in the component are mutually reachable, i.e., node ‘a’ reaches node ‘b’ and node ‘b’ reaches node ‘a’; (Fig. 2a). The use of time-limited reports allowed us to take full advantage of group differences in verbosity, which is directly measured by E.

Next, 1000 random graphs were created by preserving the same nodes and amount of edges, but shuffling word sequences (Fig. 2b). The *z*-scores of the original graph connectedness relative to the random graph distributions (LCCz and LSCz) were then calculated to estimate the degree of randomness of each graph (Fig. 2c). The purpose of this analysis was to formally verify whether structural aspects of thought disorder could be quantified by measuring the similarity of verbal reports to randomized speech. In this way, structural speech disorganization was mathematically defined as similarity of the verbal reports to random graphs: if there is a mathematical structure that determines a specific word sequence in the speech graph, shuffling word order will disrupt this pattern and LCC/LSC will change. As the comparison to random graphs distribution already kept strictly the same number of words in the graph, the verbosity difference is already controlled.

Negative image reports from schizophrenia subjects showed random-like connectedness (i.e., difference from random graph distribution smaller than two standard deviations) more frequently than reports from the Control group (64% of schizophrenia group vs. 5% of Control group, Chi-square test *p* = 0.0002; Fig. 2d and e). Reports from Bipolar subjects showed intermediate random-like connectedness (30%; Fig. 2d and e).

The illustrative examples shows that subjects from the schizophrenia group report a story based on a negative image recently seen with a less connected structure (fewer edges, smaller LCC and LSC), more similar to what would be expected from random graphs with the same words (LCCz and LSCz within 2 standard deviations) than other groups (Fig. 2f).

**Fig. 3:**
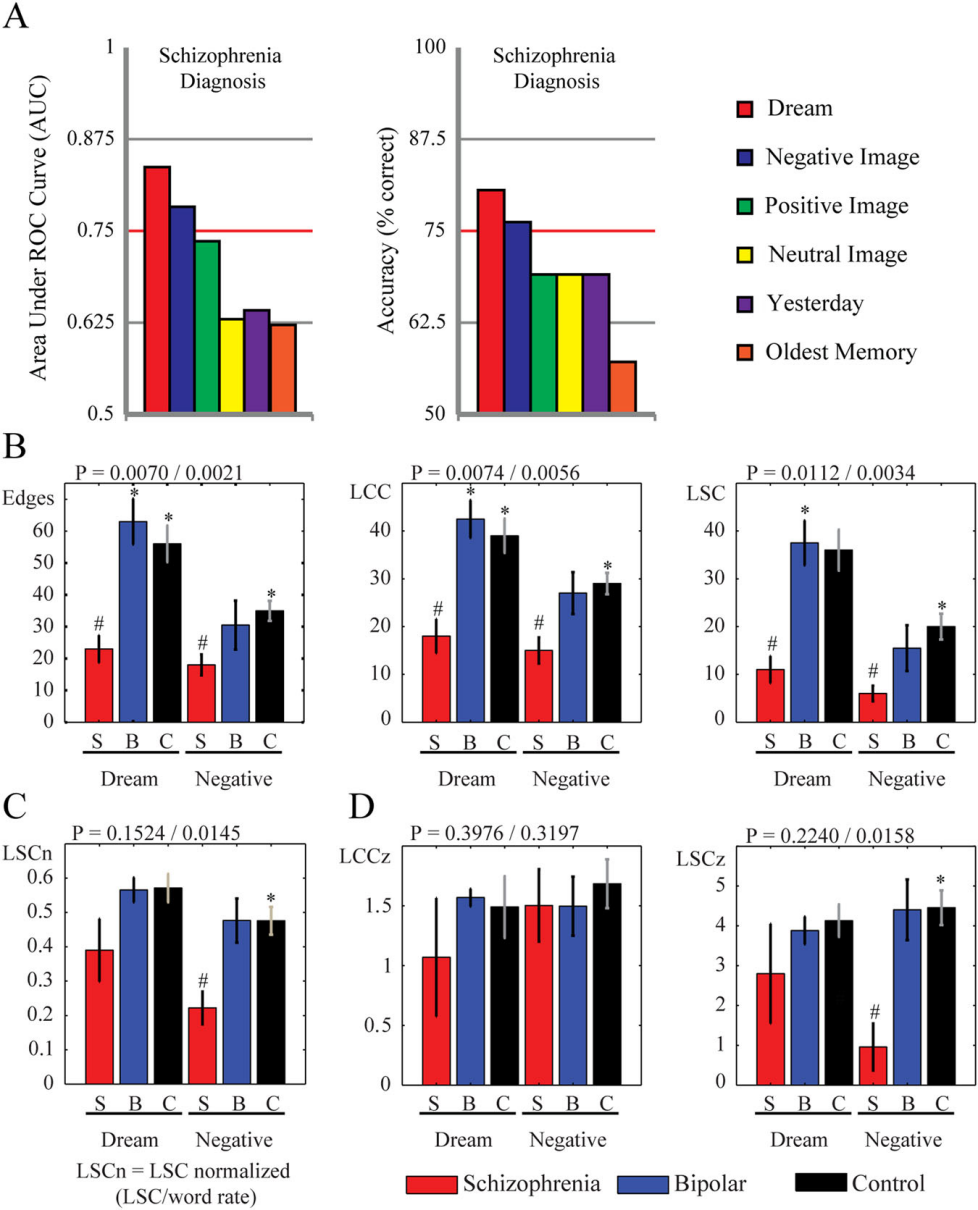
Comparison of different methods for eliciting informative reports in terms of their discrimination performance for schizophrenia. Dream and Negative image reports are more discriminating than long-term memories. **a** Schizophrenia diagnostic classification using 5 connectedness attributes (E, LCC, LSC, LCCz, and LSCz) using 6 time-limited memory reports. Only dream and negative image reports classified schizophrenia group vs. Bipolar and Control group with AUC > 0.75 and accuracy > 75%. **b** Connectedness attributes from dream and negative image reports compared between groups. **c** LSC normalized by word rate from dream and negative image reports compared between groups **d** The *z*-scores of the original graph connectedness relative to the random graph distributions (LCCz and LSCz) from dream and negative image reports compared between groups. *Bar* plots indicate of median values and *error bars* indicate standard error of the mean (s.e.m); Kruskal–Wallis tests: *p* value for dream/negative image reports indicated in each title; Wilcoxon–Ranksum tests (Bonferroni corrected for 8 comparisons (4 comparison for each 2 memory reports—SxB, SxC, Sx(B + C), and BxC)): # means *p* < 0.0063—Schizophrenia vs. Bipolar and Control groups, asterisk means *p* < 0.0063—Schizophrenia vs. Bipolar or Control groups

Using 5 connectedness attributes from each memory report as inputs to a binary classifier, only dream reports and negative image reports allowed to discriminate the schizophrenia diagnosis against other conditions (Bipolar disorder or Control), with area under the receiver operating characteristic curve (AUC) >0.75 and accuracy (Acc) >75% correct (Fig. 3a, Supplementary Table 1). Dream reports yielded better classification than negative image reports (Fig. 3a, Supplementary Table 1). However, some subjects were unable to recall a dream during their first interview (Fig. 1a), so that 36% of the schizophrenia group (*N* = 4), 20% of the Bipolar group (*N* = 2), and none of the Control subjects failed to recall a dream. For this reason, further analyses used only these 2 report types.

Non-parametric statistical tests were chosen to assess the dataset, which was not normally distributed but had homogeneous variances (Supplementary Table 2). As predicted, schizophrenia subjects produced less connected reports than subjects from other groups, with fewer edges and smaller connected components (Figs 2f and 3b, Supplementary Table 2). In the control group there were no gender-related differences for any graph attribute from any kind of report (Supplementary Table 2). When verbosity was controlled by dividing E, LCC and LSC by word rate (amount of words produced in the 30 s limited reports), negative image reports still showed significant LSC differences (Fig. 3c; Kruskal–Wallis test *p* = 0.0145; LSC/word rate Schizophrenia < Control with *p* = 0.0033 and Schizophrenia < [Control + Bipolar] with *p* = 0.0055, Wilcoxon Ranksum test). Also negative image reports showed higher similarity with random connectedness (LSCz were smaller for Schizophrenia group compared to Control group, Wilcoxon Ranksum test *p* = 0.0033, and smaller than Control + Bipolar groups, Wilcoxon Ranksum test *p* = 0.0060, Fig. 3d, Supplementary Table 2).

In further agreement with our prediction, connectedness attributes were anti-correlated with the PANSS negative subscale for dream and negative images reports (Supplementary Table 3), and there were no significant correlations between any connectedness attribute and the potential confounding factors age, years of education or chlorpromazine equivalent dose (Supplementary Table 4). Interestingly, connectedness attributes from negative image reports were more frequently correlated with negative symptoms than connectedness attributes from dream reports (Supplementary Table 3).

Next we combined all the connectedness attributes that showed significant differences among the groups. Multiple linear correlations were calculated between total PANSS negative subscale scores and seven attributes from both kinds of memory report (E, LCC, LSC, and LSCz from negative image reports; E, LCC, and LSC from dream reports), or four attributes exclusively from negative image reports, or three attributes exclusively from dream reports. Since all these parameters are to some extent correlated with verbosity,^9^ collinearity among attributes is a serious concern. To address this issue we performed a collinearity diagnosis and sequentially excluded the most collinear variables until a combination without collinearity was reached.

**Fig. 4:**
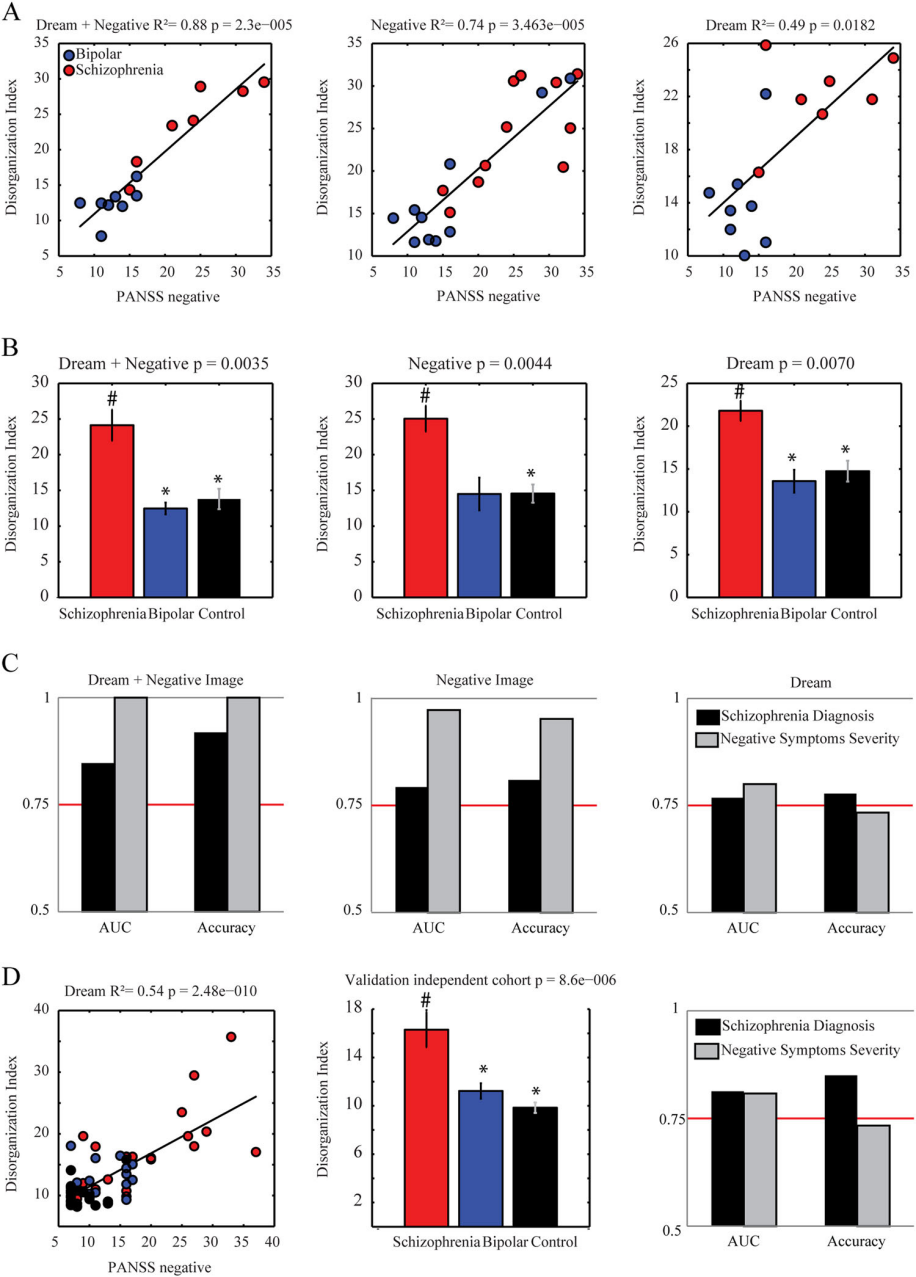
Disorganization Index classifies negative symptoms severity and schizophrenia diagnosis 6 months in advance. **a** Multiple linear correlation between PANSS negative subscale vs. Disorganization Index from dream + negative image reports, from negative image reports, or from dream reports (*R* ^2^ and *p* value indicated on title; linear coefficients used to calculate Disorganization Index on Results). **b** Bar plot of the mean and standard error of Disorganization Index from dream + negative image reports, from negative image reports, or from dream reports for diagnostic groups (schizophrenia in *red*, bipolar in *blue* and control in *black*; *bar* plots indicate of median values and *error bars* indicate s.e.m; Kruskal–Wallis tests (Bonferroni corrected for 6 comparisons (2 memory reports asterisk 3 groups)): *p* value indicated in each title; # indicates *p* < 0.0063—Schizophrenia > Bipolar and Control groups; asterisk indicates *p* < 0.0063—Schizophrenia > Bipolar or Control groups). **c** Classification quality using only Disorganization Index from dream + negative image reports, from negative image reports, or from dream reports (measured by AUC and Accuracy—classification of schizophrenia diagnosis 6 months in advance (*black*); Negative Symptom Severity measured by PANSS negative subscale (*gray*). **d** Validation of the Disorganization Index using dream reports from an independent cohort of chronic psychotic patients.^9^ Multiple linear correlation between PANSS negative subscale vs. Disorganization Index (*R* ^2^ and *p* value indicated on title; linear coefficients used to calculate Disorganization Index on Results), statistical comparison (schizophrenia in *red*, bipolar in *blue* and control in *black*; Kruskal–Wallis tests: *p* value indicated in each title; # indicates *p* < 0.0063—Schizophrenia > Bipolar and Control groups; asterisk indicates *p* < 0.0063—Schizophrenia > Bipolar or Control groups) or classification quality (measured by AUC and Acc—classification of schizophrenia diagnosis 6 months in advance (*black*); Negative Symptom Severity measured by PANSS negative subscale (*gray*))

The combination of non-collinear connectedness attributes from both kinds of reports explained nearly all the variance in total negative symptoms (Fig. 4a; *R* ^2^ = 0.88, *p* < 0.0001, observed power = 1), while using only negative image reports explained substantially less (Fig. 4a; *R* ^2^ = 0.74, *p* < 0.0001, observed power = 0.9998), and using only dream reports even less (Fig. 4a; *R* ^2^ = 0.49, *p* = 0.0182, observed power = 0.8764). The following equations defined “Disorganization Indices” for either a combination of dream and negative image reports, or separately for negative image or dream reports:

$$\begin{array}{ccccc}{\rm{Disorganization}}\,&\kern-8pt {\rm{Index}}\,({\rm{Negative}}+{\rm{Dream}})=30.78 \hfill  \\ & +{\rm{LSC}}\_{\rm{negative}}\times (0.015)+\,{\rm{LSCz}}\_{\rm{negative}} \\ & \times (-2.33)+{\rm{LCC}}\_{\rm{dream}}\times (-0.20)\hfill \\ \end{array}$$

$$\begin{array}{ccccc}{\rm{Disorganization}}&\kern-7pt {\rm{Index}}({\rm{Negative}})=31.43\hfill  \\ & +\,{\rm{LCC}}\times (-0.30)+{\rm{LSC}}\times (0.08)+{\rm{LSCz}}\times (-2.12)\\ \end{array}$$

$$\begin{array}{ccccc}{\rm{Disorganization}}\,&\kern-8pt {\rm{Index}}({\rm{Dream}})=27.82\hfill  \\ & +\,{\rm{LCC}}\times (-0.32)+{\rm{LSC}}\times (-0.012)\\ \end{array}$$

The schizophrenia group showed a higher Disorganization Index than the other groups using both kinds of reports (Kruskal–Wallis *p* = 0.0035, Fig. 4b, Supplementary Table 5), using only negative image reports (Kruskal–Wallis *p* = 0.0044, Fig. 4b, Supplementary Table 5), or using only dream reports (Kruskal–Wallis *p* = 0.0070, Fig. 4b, Supplementary Table 5). The Disorganization Index from both kinds of reports correctly classified the schizophrenia diagnosis with accuracy higher than 90%, and also classified the negative symptoms severity perfectly (Fig. 4c, Table 2). The Disorganization Indices calculated exclusively from negative image reports or from dream reports were also discriminative, but less so (Fig. 4c, Table 2).

**Table 2:** Classification quality of sorting Schizophrenia patients from others subjects, or sorting between low and high negative symptom severity, using the Disorganization Index obtained from dream + negative image reports, negative image reports, or dream reports only

Disorganization Index	Classification	Sensitivity	Specificity	Precision	Recall	F-measure	AUC	Accuracy
Dream + Negative Recent-onset Sample	S × (B + C)	0.92	0.76	0.91	0.92	0.91	0.85	91.67
	High × Low	1.00	1.00	1.00	1.00	1.00	1.00	100.00
Only Negative Recent-onset Sample	S × (B + C)	0.81	0.64	0.80	0.81	0.80	0.79	80.95
	High × Low	0.95	0.95	0.96	0.95	0.95	0.97	95.23
Only Dream Recent-onset Sample	S × (B + C)	0.78	0.62	0.80	0.78	0.79	0.77	77.78
	High × Low	0.73	0.67	0.73	0.73	0.73	0.80	73.33
Dream—Chronic Sample	S × (B + C)	0.85	0.78	0.85	0.85	0.85	0.81	85.00
	High × Low	0.73	0.62	0.72	0.73	0.72	0.81	73.33

The last row shows an independent validation of the Disorganization Index calculated for dream reports of a chronic psychotic sample.^9^ S × (B + C) indicates that the classification was performed between the Schizophrenia group (S) vs. the sum of Bipolar and Control groups (B + C)

In order to understand how much of the information in the Disorganization Index is actually due to verbosity differences, we verified that all the 3 Disorganization Indices were correlated with word rate (Spearman correlation between word rate and Disorganization Index from dream and negative image reports: Rho = −0.67, *p* = 0.0059; exclusively from negative image reports: Rho = −0.84, *p* < 0.0001; exclusively from dream reports: Rho = −0.96, *p* < 0.0001), but the correlation between the Disorganization Indices and negative symptoms remained significantly different when adjusted for word rate (adjusted Spearman correlation by word rate between PANSS negative subscale and index from dream and negative image reports: Rho = 0.84, *p* = 0.0001; index from negative image reports only: Rho = 0.57, *p* = 0.0087), except for the Disorganization Index calculated exclusively from dream reports (Rho = 0.18, *p* = 0.5346; Bonferroni correction for 3 comparisons, α = 0.0167).

Importantly, there was an 82% overlap between the schizophrenia group and the psychotic patients that presented high scores in the PANSS negative subscale. Also, there was no significant Spearman correlation between any Disorganization Index and the potential confounding factors age, years of education and chlorpromazine equivalent dose (Supplementary Table 6), neither did these factors disrupt the Spearman correlation between Disorganization Index and PANSS negative subscale when considered as adjustment (Supplementary Table 6), except for the effect of medication dose in the correlation between negative symptoms and the Disorganization Index calculated exclusively from dream reports (Supplementary Table 6). This could be due to a weaker relationship with negative symptoms in these reports, or to a smaller sample of dream reports in comparison to negative image reports, since not all subjects were able to recall dreams.

To validate the method in an independent cohort, the same strategy was applied to dream reports of a previously collected sample of chronic psychotic patients and controls,^9^ which was not normally distributed and had homogeneous variances (Fig. 1b, Supplementary Table 5). There was a similar multiple correlation of connectedness attributes with negative symptoms (*R* ^2^ = 0.54, *p* < 0.0001, observed power = 1), which after the exclusion of collinear variables led to a Disorganization Index = 93.91 + E × (−3.08) + LSC × (0.21). The statistical differences among the groups resembled those found in the recent-onset psychosis sample (Kruskal–Wallis *p* < 0.0001, Fig. 4d, Supplementary Table 5), and the Disorganization Index was also quite informative of the schizophrenia diagnosis and the severity of negative symptoms (Fig. 4d, Table 2). It was also possible to validate diagnosis and symptom severity classification using the index calculated from a sample to another sample (Supplementary Table 7).

Finally, in both the recent-onset and chronic psychosis samples, there were no statistically significant differences between the Bipolar and the Control groups for any connectedness attribute, either in isolation or combined into the Disorganization Index (Supplementary Table 2 and 5).

## Discussion

One of the promises of computational psychiatry is to provide quantitative phenotyping of relevant psychiatric symptoms.^5–7, 17^ Here we showed that speech graph analysis allows for the structural quantification of formal thought disorder, mathematically defined by the linear combination of connectedness graph attributes and their degree of similarity to randomly generated graph attributes. This procedure offers unbiased and precise numbers to what was previously only described by words. While the results can be partially explained by verbosity differences, especially with regard to dream reports, subjects from the schizophrenia group showed smaller LSC even after controlling for verbosity (either normalizing attributes by word rate, or comparing to random graphs with the same amount of words). Furthermore, verbosity could not explain the relationship between negative symptoms and Disorganization Indices, except for the Index calculated exclusively from dream reports.

The four hypotheses raised were verified. Dream and negative image short-term memory reports could be used—and their combination was optimal—to discriminate the schizophrenia diagnosis 6 months in advance. Connectedness attributes from dream reports were most discriminative of schizophrenia, with better performance than connectedness attributes from waking reports.^9^ However, the difficulty shown by some subjects to recall dreams was a practical clinical concern that could be circumvented using short-term memory reports based on affective images.^14^ As predicted, short-term memory reports were more informative than long-term memory reports (“yesterday” or “oldest” memories).

The results show that connectedness is often impaired in schizophrenia patients, to the point of being undistinguishable from random values in 64% of the subjects in this group. The estimation of the randomness degree of connectedness provides a quantitative measurement of though disorder at the structural level. Such structural disorganization is likely exacerbated in subjects with advanced cognitive impairment, as in patients with the psychopathological symptom “word salad”.^18^ Note in this regard that connectedness as measured by graph analysis does not directly estimate semantic relationships, although we have recently reported a significant correlation (*R* = −0.4) between LSC and semantic incoherence.^19^ Furthermore, the psychotic subjects studied here were not expressing full-fledged “word salad”, understood as extreme speech disorganization at both the structural and semantic levels, which rarely occurs in early-course psychosis. While the analogy with “word salad” must be taken with caution, the quantitative method to assess thought disorganization presented here has major potential for revealing early signs of thought disorder, measurable even before semantic incoherence becomes clinically evident.

The results also confirmed that connectedness is negatively correlated with negative symptom severity. A linear combination of connectedness attributes explained nearly all the variance of the negative symptoms severity, and reached high classification accuracy for negative symptom severity (100% when combining both reports) and of schizophrenia diagnosis 6 months in advance. There was a very high overlap (82%) between the schizophrenia diagnosis and high scoring in the PANSS negative subscale, but overall the accuracy was better for negative symptoms severity than for DSM diagnosis. This raises the point that precise behavioral measurements are more likely to describe symptomatology than standard diagnosis.^20^ Importantly, it was possible to correctly classify schizophrenia diagnosis and negative symptom severity using the Disorganization Index from dream reports of an independent cohort of chronic psychotic patients and control subjects interviewed years before the present study.^9^

Of note, the Bipolar and Control groups could not be differentiated using neither connectedness attributes nor the Disorganization Index. Semantic computational strategies, rather than the structural approach chosen here, may be better to predict psychotic breaks during prodromal stages,^11^ or to differentiate patients with Bipolar Disorder from healthy controls.^21^

Our study has some limitations worth mentioning. First, to obtain sound psychopathological boundaries for the Disorganization Index, i.e., more reliable estimations of the linear combination coefficients, it will be necessary to investigate a larger sample better matched for gender and educational level, with multiple researchers scoring negative symptoms at high inter-rater reliability. Second, the sample sizes of the present study were based on the prevalence of schizophrenia. While the main results reached very high observed power, future studies should also consider statistical power a priori when planning sample sizes. Third, the findings must be replicated with native speakers of other languages to assert their general applicability. Fourth, the medications taken by the schizophrenia and Bipolar groups could not be rigorously matched due to treatment differences between the pathologies, and to the non-interventional experimental design. Indeed, we found an important impact of adjusting for medication dose in the correlation of negative symptoms with the Disorganization Index calculated exclusively from dream reports, and therefore medication should be better controlled in future studies. Fifth, the duration of psychotic symptoms before the first clinical interview was estimated by interviews with families and patients, and therefore was not precisely measured.^22^ Sixth, a longitudinal prodromal evaluation is in order to describe how graph attributes progress over time in relation to clinical evolution, and how sensitive these attributes are to medication changes.

Beyond these limitations, our study exemplifies how computational strategies can precisely measure important psychiatric symptoms using a naturalistic approach that mathematically characterizes what psychiatrists have for decades subjectively described in clinical practice. Graph analysis is a fast and low-cost tool for complementary psychiatric evaluation. The recording of two time-limited memory reports takes ~3 min, audio transcription takes ~10 min, and data processing from text transcript to graph analysis is nearly instantaneous.^9^ Whenever a patient fails to recall a dream, it is still possible to calculate an accurate Disorganization Index using only a negative image report. The method presented is directly based on the psychopathological description of formal though disorder in schizophrenia, shows substantial discriminative power, and represents a successful translation of basic science into applied technology able to improve clinical evaluation.

## Methods

### Study design

This prospective study recruited patients interviewed during first clinical contact for recent-onset psychosis in a public child psychiatric clinic (CAPSi) in Natal, RN, Brazil, from August 2014 to July 2015. All patients had the initial diagnosis of psychotic episode under evaluation, and were followed up for 6 months by an interdisciplinary clinical team, who evaluated information from different sources including family, school environment, clinical assessment, and exams. After 6 months the cases were discussed by the team and disease diagnosis was established according to DSM IV criteria (applying SCID).^23^ This reference standard was chosen for compatibility with previous studies using graph analysis to investigate psychosis.^9, 10^ After the psychosis sample was collected, well-matched controls were recruited on nearby public schools. The parameters matched were age, sex, socio-economic status, and educational level. Matching was facilitated by the fact that Brazilian public schools have high levels of age-grade delay.^24^ Psychotic and control groups were collected as convenience samples. Data analysis began after the entire sample was collected and all patients had finished follow-up (the index was not available during the clinical follow-up and diagnosis was not available during the speech recording). The method was validated on dream reports from an independent cohort of chronic psychotic subjects and matched controls recruited at convenience samples at Hospitals Onofre Lopes and João Machado (in Natal, RN, Brazil) between February 2008 and October 2012.

Sample sizes were based on Brazil’s prevalence^25^ of schizophrenia using the following equation:

$${N}={{Z}}^{2}{P}(1-{P})/{{d}}^{2}$$

(*Z* statistic for a level of confidence = 1.96, considering 95% of confidence interval; *P* was the prevalence, considered 0.57%,^26^ and *d* was the precision = 0.05). The estimated sample size was *N* = 9. We doubled the value of *N*, considering that some individuals would be expected to have Bipolar disorder diagnosis in the end of the follow-up.

### Participants

Study approved by the UFRN Research Ethics Committee (permit # 742–116 for recent-onset psychosis sample, permit #102/06-98244 for chronic psychosis sample). Pre-established exclusion criteria comprised having any neurological symptom, or having drug-related disorders. Twenty-two patients undergoing recent-onset psychosis (Table 1) were recruited during first psychiatric interview and followed up for 6 months to establish diagnoses. Inclusion criterion was to be seeking treatment for psychotic symptoms for the first time (maximum duration of two years as reported by patient and family members). One patient was excluded after epilepsy diagnosis. Twenty-one healthy control subjects matched by age, sex, and education were interviewed during regular class time in public schools of Natal, RN, Brazil (Table 1). An additional exclusion criterion for the Control group was not having any psychiatric symptom or diagnosis, as assessed during family member interviews.

The independent cohort comprised subjects diagnosed according to DSM-IV^9^ with schizophrenia (*n* = 20), or Bipolar Disorder (*n* = 20), as well as subjects without psychosis. Participants and legal guardians provided written informed consent.

### Protocol

Subjects were submitted to an audio-recorded interview that consisted of requests for six time-limited memory reports. In order to minimize inter-subject differences in word count, each report was limited to 30 s. Whenever the subject spontaneously stopped the report, he/she was stimulated to keep talking by way of general instructions like “please, tell me more about it”. When the report reached the 30-s limit, the interviewer interrupted the report saying “ok”. The interview began with a request to produce a “dream report” (either recent or remote). Next, the “oldest memory report” was obtained by requesting the subjects to report the most remote memory they could access at that moment. Then the subjects were requested to report on their previous day (“yesterday report”), and finally they were exposed to three images presented on a computer screen, comprising a “highly negative image”, a “highly positive image” and a “neutral image” from the IAPS database^16^ of affective images, previously tested in children^16^ and psychotic subjects.^27^ Subjects were instructed to pay attention to each image for 15 s and then report an imaginary story based on it. The entire memory report protocol took up to 10 min to be completed. Subjects undergoing recent-onset psychosis were then evaluated psychiatrically using the psychometric scale PANSS^28^ composed of three subscales (positive, negative, and general). The negative subscale measured seven symptoms: Blunted affect (N1), Emotional withdrawal (N2), Poor rapport (N3), Passive/apathetic social withdrawal (N4), Difficulty in abstract thinking (N5), Lack of spontaneity and flow of conversation (N6), Stereotyped thinking (N7).^28^ Only one researcher performed PANSS scoring (NBM), and all the psychometric evaluations were completed during the data collection, and therefore prior to speech graph analysis.

### Graph measures

The search for a discriminative index of connectedness was exploratory, and for that we tested six different kinds of memory reports. Memory reports were transcribed and represented as graphs in which each word was represented as a node, and the temporal sequence between consecutive words was represented by directed edges (Fig. 2a) using the software *SpeechGraphs* (http://www.neuro.ufrn.br/softwares/speechgraphs) (code freely available).^9^ Three connectedness attributes were calculated: Edges (E), which measures the amount of links between words; LCC, which measures the amount of nodes in the largest component in which each pair of nodes has a path between them; and LSC, which counts the amount of nodes in the largest component in which each pair of nodes has a mutually reachable path, i.e., node “a” reaches node “b” and node “b” reaches node “a” (Fig. 2a).

We compared each memory report graph to 1000 random graphs built with the same nodes and number of edges, but with a random shuffling of the edges that amounts to shuffling words (Fig. 2b). Next we estimated the LCC and LSC *z*-scores between each original graph and the corresponding random graph distribution (Fig. 2c). These normalized attributes were termed LCCz and LSCz. Formally, LCCz = (LCC—LCCmr) / LCCsdr and LSCz = (LSC—LSCmr) / LSCsdr, with LCCmr and LSCmr corresponding respectively to mean LCC and LSC values in the random graph distributions; likewise, LCCsdr and LSCsdr denote the standard deviation of LCC and LSC from the random graph distribution. A graph was considered random-like when its connectedness attributes fell within two standard deviations from the mean of the random distribution (Fig. 2d).

### Analyses

All the statistical analyses used Matlab software. To avoid over-fitting and better combine the most informative connectedness attributes, we first applied five connectedness attributes (E, LCC, LSC, LCCz, and LSCz) from each memory report as inputs to a Naïve Bayes classifier with cross-validation (10-fold) implemented with Weka software,^29^ and trained for the binary choice between the schizophrenia group vs. the sum of Bipolar and Control groups, using as golden standard the diagnostic reached after 6 months of follow-up. Classification quality was assessed using Accuracy (Acc, percentage of correctly classified subjects) and AUC. A threshold of Acc = 75% correct or AUC = 0.75 was established in order to consider a memory report informative (Fig. 3a, Supplementary Table 1). Using Spearman correlations, we related each connectedness attribute from each informative memory report to the PANSS negative subscale (Supplementary Table 3), and compared the groups applying Kruskal–Wallis and two-sided Wilcoxon–Ranksum test (Fig. 3b, Supplementary Table 2). All statistical analyses were corrected for multiple comparisons (Bonferroni). Normality and variance homogeneity were assessed by the Kolmogorov–Smirnov and Levene tests, respectively. As the sample distribution was not normal, we used only non-parametric statistical tests.

To calculate the Disorganization Index, we began by selecting only the connectedness attributes that presented any significant statistical difference between groups after Bonferroni correction. Following the selection of these most informative connectedness attributes, they were combined and correlated with the total score of the PANSS negative subscale using multilinear regression (Fig. 4a). Multicollinearity diagnosis was performed to guarantee a non-collinear combination. Variables with the largest variance decomposition proportion whenever the conditioning index was higher than ten were sequentially excluded until a non-collinear combination was reached. Attribute coefficients were then extracted and this linear combination was used to create the Disorganization Index (equation described in the Results Session). Since the sample size was planned based on the prevalence of schizophrenia, we estimated the statistical power a posteriori (observed power) to guarantee regression results with power higher than 0.80.^30^ We also verified whether the Disorganization Index differed between the groups using Kruskal–Wallis and two-sided Wilcoxon Ranksum tests with Bonferroni correction for four comparisons: Schizophrenia vs. [Bipolar + Control], Schizophrenia vs. Bipolar, Schizophrenia vs. Control, Bipolar vs. Control (*α* = 0.0125; Fig. 4b, Supplementary Table 4). Normality and variance homogeneity were assessed by the Kolmogorov–Smirnov and Levene tests, respectively. Partial Spearman correlations to control for confounding factors were implemented using the Matlab code partialcorr.

To verify whether the Disorganization Index could classify the schizophrenia diagnosis using only connectedness attributes from memory reports recorded during the first psychiatric interview, a binary classifier Naïve Bayes^29^ with 10-fold cross-validation was used to sort the patients that 6 months later received the schizophrenia diagnosis from other groups. To verify whether the Disorganization Index could correctly sort patients with severe negative symptoms from those with milder negative symptomatology, the samples were divided in two subsamples with high (more than the median) and low (less or equal the median) scores of total PANSS negative subscale. The cutoff was the PANSS median of the entire group of psychotic patients (Schizophrenia + Bipolar). The median PANSS value was 16. Next we verified whether the Naïve Bayes classifier was able to classify both samples using only the Disorganization Index. Classification quality was verified by measuring true positive rate (sensitivity), true negative rate (1-specificity), precision, recall, f-measure, AUC and Acc (Table 2).

The same strategy to obtain a Disorganization Index was validated in a previously collected sample of dream reports from chronic psychotic subjects and matched controls.^9^ As this previous protocol was not time-limited, verbosity differences were controlled using average graph attributes from 30-word graphs (see ref. 9 for details). Also a validation of the index across samples were calculated (applying the index calculated for dream reports from recent-onset sample to chronic psychotic data, and index calculated for chronic psychotic sample for recent-onset psychosis data). Classification accuracy for schizophrenia diagnosis and negative symptom severity was verified using Naïve Bayes classifiers (Supplementary Table 7). All the graph attribute measurements used in the current study are available as Supplementary Information (Supplementary Tables 8, 9, and 10). For research purposes only, all the raw transcribed data are available in our webpage (http://neuro.ufrn.br/multiusuario/cadastramento/?page_id=19).

## Electronic supplementary material

Supplementary Table 1

Supplementary Table 2

Supplementary Table 3

Supplementary Table 4

Supplementary Table 5

Supplementary Table 6

Supplementary Table 7

Supplementary Table 8

Supplementary Table 9

Supplementary Table 10

Supplementary Information
