# Supplementary material for: Thought disorder measured as random speech structure classifies negative symptoms and schizophrenia diagnosis 6 months in advance
Source: NPJ Schizophr. 2017 Apr 13;3:18. doi: 10.1038/s41537-017-0019-3 (PMC5441540; doi:10.1038/s41537-017-0019-3)
Supplement: Supplementary file 1 — Supplementary Table 1 [file 41537_2017_19_MOESM1_ESM.pdf]

Supplementary Table 1: Classification quality to classify Schizophrenia group from others subjects using a Naïve Bayes classifier with all 5 connectedness attributes (E, LCC, LSC, LCCz, LSCz) from different time-limited memory reports.

Groups	Sensitivity	Specificity	Precision	Recall	F-Measure	AUC	Accuracy
**Dream**	0.81	0.85	0.87	0.81	0.82	0.84	80.56
**Negative**	0.76	0.68	0.78	0.76	0.77	0.78	76.19
**Positive**	0.69	0.77	0.79	0.69	0.71	0.74	69.05
**Neutral**	0.69	0.71	0.76	0.69	0.71	0.63	69.05
**Yesterday**	0.69	0.54	0.70	0.69	0.70	0.64	69.05
**Oldest**	0.57	0.56	0.66	0.57	0.60	0.62	57.14
