# Supplementary material for: Thought disorder measured as random speech structure classifies negative symptoms and schizophrenia diagnosis 6 months in advance
Source: NPJ Schizophr. 2017 Apr 13;3:18. doi: 10.1038/s41537-017-0019-3 (PMC5441540; doi:10.1038/s41537-017-0019-3)
Supplement: Supplementary file 2 — Supplementary Table 2 [file 41537_2017_19_MOESM2_ESM.pdf]

Supplementary Table 2: Statistical comparison of connectedness attributes (E, LCC, LSC, LCCz, LSCz) between diagnostic groups (Schizophrenia = S, Bipolar = B, Control = C). KS test rejects normal distribution of all samples (Bonferroni corrected for 2 comparisons, $p < 0.0250$ in bold), Levene's test verifies variance homogeneity (Bonferroni corrected for 2 comparisons, $p < 0.0250$ in bold). Kruskal-Wallis test (SxBxC, Bonferroni corrected for 2 comparisons (2 memory reports), $p < 0.0250$ in bold); Wilcoxon-Ranksum test (SxB, SxC, SxB+C, BxC; Bonferroni corrected for 6 comparisons (4 comparison for each memory reports), $p < 0.0063$ in bold). Statistical comparison using Wilcoxon-Ranksum test between male x female in Control group showed no difference (Bonferroni corrected for 2 comparisons (2 memory reports), $p < 0.0250$ in bold)).

**KS test**		**E**	**LCC**	**LSC**	**LCCz**	**LSCz**
**Dream**	p value	**3.60E-33**	**3.60E-33**	**5.45E-25**	**4.59E-16**	**1.01E-22**
	h	1	1	1	1	1
**Negative**	p value	**2.39E-38**	**9.68E-37**	**2.42E-27**	**2.66E-16**	**4.70E-21**
	h	1	1	1	1	1
**Levene's Test**		**E**	**LCC**	**LSC**	**LCCz**	**LSCz**
**Dream**		0.0804	0.1266	0.0349	0.4017	0.0603
**Negative**		0.1264	0.3544	0.095	0.8135	0.7008
**Kruskal-Wallis**		**E**	**LCC**	**LSC**	**LCCz**	**LSCz**
**Dream**	S x B x C	**0.0070**	**0.0074**	**0.0112**	0.3976	0.2240
**Negative**	S x B x C	**0.0021**	**0.0056**	**0.0034**	0.3197	**0.0158**
**Wilcoxon Ranksum**		**E**	**LCC**	**LSC**	**LCCz**	**LSCz**
**Dream**	SxB	**0.0056**	**0.0031**	**0.0040**	0.1893	0.1893
	SxC	**0.0042**	**0.0045**	0.0079	0.2652	0.1239
	Sx(B+C)	**0.0021**	**0.0019**	**0.0031**	0.1872	0.1013
	BxC	0.5418	0.8640	0.8642	0.9029	0.5419
**Negative**	SxB	0.0081	0.0181	0.0205	0.3418	0.1300
	SxC	**0.0009**	**0.0022**	**0.0009**	0.1421	**0.0033**
	Sx(B+C)	**0.0005**	**0.0015**	**0.0008**	0.1446	**0.0060**
	BxC	0.7997	0.6719	0.8823	0.7513	0.4856
**Wilcoxon Ranksum**		**E**	**LCC**	**LSC**	**LCCz**	**LSCz**
**Dream**	Male x Fem	0.6959	1.0000	0.6440	0.9717	0.9151
**Negative**	Male x Fem	0.6439	0.3922	0.5936	0.2707	0.2707
