# Supplementary material for: Thought disorder measured as random speech structure classifies negative symptoms and schizophrenia diagnosis 6 months in advance
Source: NPJ Schizophr. 2017 Apr 13;3:18. doi: 10.1038/s41537-017-0019-3 (PMC5441540; doi:10.1038/s41537-017-0019-3)
Supplement: Supplementary file 3 — Supplementary Table 3 [file 41537_2017_19_MOESM3_ESM.pdf]

Supplementary Table 3: Spearman correlation between connectedness attributes (E, LCC, LSC, LCCz, LSCz) and negative symptoms measured by PANSS (total negative subscale, N1, N2, N3, N4, N5, N6, N7), using dreams or negative image reports. Showed R, and p values (significant results in bold after Bonferroni correction for 80 comparisons – 5 attributes * 2 reports * 8 symptoms, p < 0.0006).

Dream Reports	E		LCC		LSC		LCCz		LSCz	
PANSS Negative Subscale	Rho	p	Rho	p	Rho	p	Rho	p	Rho	p
Total	-0.69	0.0046	-0.69	0.0042	-0.65	0.0089	-0.41	0.132	-0.16	0.5654
N1	-0.71	0.0028	-0.71	0.0031	-0.72	0.0026	-0.34	0.2121	-0.23	0.4098
N2	-0.85	**0.0001**	-0.8	**0.0003**	-0.76	0.0009	-0.39	0.1463	-0.2	0.4775
N3	-0.57	0.0279	-0.57	0.0279	-0.56	0.0286	-0.25	0.3755	-0.11	0.6962
N4	-0.56	0.0317	-0.48	0.0724	-0.4	0.1392	-0.11	0.6852	0.33	0.2355
N5	-0.44	0.0978	-0.49	0.0634	-0.47	0.0757	-0.39	0.1459	-0.46	0.0836
N6	-0.6	0.0192	-0.6	0.0183	-0.57	0.0281	-0.44	0.0988	-0.2	0.4774
N7	0.63	0.0126	0.64	0.0101	0.6	0.0184	0.28	0.3200	0.33	0.2342
Negative Image Reports	E		LCC		LSC		LCCz		LSCz	
PANSS Negative Subscale	Rho	p	Rho	p	Rho	p	Rho	p	Rho	p
Total	-0.81	**0.0000**	-0.85	**0.0000**	-0.81	**0.0000**	-0.7	**0.0005**	-0.77	**0.0001**
N1	-0.78	**0.0000**	-0.8	**0.0000**	-0.77	**0.0000**	-0.63	0.0021	-0.69	**0.0006**
N2	-0.77	**0.0000**	-0.77	**0.0001**	-0.75	**0.0001**	-0.62	0.0027	-0.67	0.0008
N3	-0.8	**0.0000**	-0.77	**0.0000**	-0.82	**0.0000**	-0.59	0.0051	-0.75	**0.0001**
N4	-0.69	**0.0006**	-0.73	**0.0002**	-0.62	0.0026	-0.69	**0.0005**	-0.57	0.0065
N5	-0.63	0.0024	-0.66	0.0011	-0.66	0.0012	-0.46	0.0364	-0.67	0.0008
N6	-0.8	**0.0000**	-0.81	**0.0000**	-0.81	**0.0000**	-0.57	0.0065	-0.73	**0.0002**
N7	0.32	0.1562	0.26	0.2543	0.24	0.2998	-0.02	0.9409	0.05	0.8288
