# Supplementary material for: Thought disorder measured as random speech structure classifies negative symptoms and schizophrenia diagnosis 6 months in advance
Source: NPJ Schizophr. 2017 Apr 13;3:18. doi: 10.1038/s41537-017-0019-3 (PMC5441540; doi:10.1038/s41537-017-0019-3)
Supplement: Supplementary file 4 — Supplementary Table 4 [file 41537_2017_19_MOESM4_ESM.pdf]

**Supplementary Table 4:** Spearman correlations between each graph attribute and confounding factors (Bonferroni corrected for 30 comparisons (2 memory reports, 3 confound factors, and 5 graph attributes, $p < 0.0017$).

| | AGE | | | | EDUCATION | | | | AP DOSE (CLPeq) | | | |
|---|---|---|---|---|---|---|---|---|---|---|---|---|
| | Dream | | Negative | | Dream | | Negative | | Dream | | Negative | |
| | rho | p | rho | p | rho | p | rho | p | rho | p | rho | p |
| **E** | -0.14 | 0.6291 | 0.17 | 0.4626 | 0.13 | 0.6455 | 0.47 | 0.0324 | -0.50 | 0.0572 | -0.42 | 0.0573 |
| **LCC** | 0.01 | 0.9746 | 0.06 | 0.7865 | 0.27 | 0.3342 | 0.40 | 0.0746 | -0.41 | 0.1270 | -0.46 | 0.0357 |
| **LSC** | -0.03 | 0.9034 | 0.35 | 0.1246 | 0.21 | 0.4563 | 0.60 | 0.0042 | -0.51 | 0.0549 | -0.30 | 0.1908 |
| **LCCz** | 0.43 | 0.1065 | -0.20 | 0.3821 | 0.32 | 0.2480 | 0.01 | 0.9529 | -0.04 | 0.8890 | -0.35 | 0.1207 |
| **LSCz** | 0.36 | 0.1879 | 0.29 | 0.1952 | 0.32 | 0.2396 | 0.50 | 0.0203 | -0.08 | 0.7798 | -0.36 | 0.1085 |
