# Supplementary material for: Thought disorder measured as random speech structure classifies negative symptoms and schizophrenia diagnosis 6 months in advance
Source: NPJ Schizophr. 2017 Apr 13;3:18. doi: 10.1038/s41537-017-0019-3 (PMC5441540; doi:10.1038/s41537-017-0019-3)
Supplement: Supplementary file 5 — Supplementary Table 5 [file 41537_2017_19_MOESM5_ESM.pdf]

**Supplementary Table 5:** Statistical comparison of Disorganization Index between diagnostic groups (Schizophrenia = S, Bipolar = B, Control = C), considering dream + negative image reports, negative image reports or dream reports and applying the Disorganization Index from dream reports to an independent cohort of chronic psychotic sample[9]. KS test rejects normal distribution of all samples (Bonferroni corrected for 4 comparisons, p < 0.0125 in bold), Levene’s test verifies variance homogeneity (Bonferroni corrected for 4 comparisons, p < 0.0125 in bold). Kruskal-Wallis test (SxBxC, Bonferroni corrected for 3 comparisons (3 Disorganization Indexes) p < 0.0167 in bold); Wilcoxon-Ranksum test (SxB, SxC, Sx(B+C), BxC; Bonferroni corrected for 8 comparisons (4 comparison for each memory reports), p < 0.0063 in bold).

| **Disorganization Index** | | **Kruskal-Wallis (p)** | **KS test (p)** | **KS test (h)** | **Levene's test (p)** |
|---|---|---|---|---|---|
| **Dream + Negative** | S x B x C | **0.0035** | **2.35E-31** | 1 | 0.0472 |
| **Negative** | S x B x C | **0.0044** | **1.89E-38** | 1 | 0.6966 |
| **Dream** | S x B x C | **0.0070** | **3.60E-33** | 1 | 0.1157 |
| **Dream - Chronic Sample** | S x B x C | **8.60E-06** | **2.87E-54** | 1 | 0.0268 |
| **Disorganization Index - Wilcoxon Ranksum test (p values)** | | | | | |
| **Dream + Negative** | SxB | **0.0006** | **Negative** | SxB | 0.0221 |
| | SxC | **0.0030** | | SxC | **0.0013** |
| | Sx(B+C) | **0.0009** | | Sx(B+C) | **0.0011** |
| | BxC | 0.7511 | | BxC | 0.7513 |
| **Dream** | SxB | **0.0037** | **Dream - Chronic Sample** | SxB | **0.0011** |
| | SxC | **0.0042** | | SxC | **0.0000** |
| | SxB+C | **0.0018** | | SxB+C | **0.0000** |
| | BxC | 0.8452 | | BxC | 0.0385 |
