# Supplementary material for: Thought disorder measured as random speech structure classifies negative symptoms and schizophrenia diagnosis 6 months in advance
Source: NPJ Schizophr. 2017 Apr 13;3:18. doi: 10.1038/s41537-017-0019-3 (PMC5441540; doi:10.1038/s41537-017-0019-3)
Supplement: Supplementary file 6 — Supplementary Table 6 [file 41537_2017_19_MOESM6_ESM.pdf]

Supplementary Table 6: Controls for confounding factor for episode psychosis group (age, educational level, and medication status). Spearman correlation between disorganization indexes and confounding factor and adjusted Spearman correlation between disorganization indexes versus negative symptoms (PANSS negative subscale), adjusted for each confounding factor (Bonferroni corrected for 6 comparisons (2 memory reports and 3 confound factors, p < 0.0083).

Confound Factors	Dream+Negative		Negative		Dream	
**Disorganization Index**	**rho**	**p**	**rho**	**p**	**rho**	**p**
Index x Age (years)	-0.12	0.6688	-0.14	0.5375	-0.01	0.9848
Index x Education (years)	-0.20	0.4639	-0.42	0.0555	-0.27	0.3380
Index x AP dose (CLPeq)	0.54	0.0385	0.32	0.1529	0.43	0.1108
**Index x PANSS negative**	**rho**	**p**	**rho**	**p**	**rho**	**p**
No Adjustment	0.92	**0.0000**	0.84	**0.0000**	0.70	**0.0038**
By Age (years)	0.92	**0.0000**	0.84	**0.0000**	0.70	**0.0054**
By Education (years)	0.91	**0.0000**	0.80	**0.0000**	0.68	**0.0070**
By AP dose (CLPeq)	0.89	**0.0000**	0.84	**0.0000**	0.61	0.0202
