# Supplementary material for: Thought disorder measured as random speech structure classifies negative symptoms and schizophrenia diagnosis 6 months in advance
Source: NPJ Schizophr. 2017 Apr 13;3:18. doi: 10.1038/s41537-017-0019-3 (PMC5441540; doi:10.1038/s41537-017-0019-3)
Supplement: Supplementary file 7 — Supplementary Table 7 [file 41537_2017_19_MOESM7_ESM.pdf]

Supplementary Table 7: Validation of coefficients across different samples. Classification quality (a Naïve Bayes classifier) of sorting Schizophrenia patients from others subjects (Diagnosis), or sorting between low and high negative symptom severity (Negative Symptoms), using the Disorganization Index obtained from dream reports of the recent-onset psychotic sample (DI1), and applied to dream reports of a chronic psychotic sample [9] (Sample 2), or Disorganization Index obtained from dream reports of chronic psychotic sample (DI2) and applied to dream reports of the recent-onset psychotic sample (Sample 1).

		**AUC**	**Accuracy (%)**
**Sample 2 in DI1**	Diagnosis	0.74	76.67
	Negative Symptoms	0.82	70.00
**Sample 1 in DI2**	Diagnosis	0.81	80.56
	Negative Symptoms	0.78	73.33
