# Supplementary material for: Thought disorder measured as random speech structure classifies negative symptoms and schizophrenia diagnosis 6 months in advance
Source: NPJ Schizophr. 2017 Apr 13;3:18. doi: 10.1038/s41537-017-0019-3 (PMC5441540; doi:10.1038/s41537-017-0019-3)
Supplement: Supplementary file 8 — Supplementary Table 8 [file 41537_2017_19_MOESM8_ESM.pdf]

Supplementary Table 8: Raw data and PANSS from recent-onset psychosis sample.

		Negative Image						Dream						PANSS							
**NoID Subjects**	**Group**	**WC**	**Edges**	**LCC**	**LSC**	**LCCz**	**LSCz**	**WC**	**Edges**	**LCC**	**LSC**	**LCCz**	**LSCz**	**Total**	**N1**	**N2**	**N3**	**N4**	**N5**	**N6**	**N7**
Subject 01	Schizophrenia	18	17	15	4	1.50	0.96	29	26	22	2	1.73	-0.57	24	5	4	3	2	5	4	1
Subject 02	Schizophrenia	32	30	24	18	1.68	4.93	11	8	6	1	-0.09	-0.54	16	3	3	1	1	5	2	1
Subject 04	Schizophrenia	24	23	20	12	1.64	4.06	41	40	35	16	2.26	3.73	15	2	2	2	3	4	1	1
Subject 05	Schizophrenia	31	29	26	7	1.93	1.63	32	31	18	17	0.81	2.80	21	3	3	3	2	6	3	1
Subject 07	Schizophrenia	5	3	3	1	0.04	-0.30							26	5	3	3	3	6	5	1
Subject 08	Schizophrenia	8	6	7	1	1.70	-0.48	24	20	18	16	1.07	8.40	31	4	4	4	6	6	5	2
Subject 09	Schizophrenia	13	7	6	1	0.40	-0.42	28	23	14	11	-1.69	3.56	25	4	5	3	5	4	3	1
Subject 10	Schizophrenia	30	27	26	8	1.93	2.58							20	5	4	4	1	1	4	1
Subject 11	Schizophrenia	32	27	19	6	0.58	0.52							33	5	5	4	5	6	5	3
Subject 03	Schizophrenia	20	18	15	8	0.77	3.33							32	6	4	5	3	6	5	3
Subject 06	Schizophrenia	8	3	2	1	-1.29	-0.25	14	9	9	1	1.33	-0.64	34	6	5	5	4	7	6	1
Subject 12	Bipolar Disorder	34	31	28	14	2.10	4.53	67	63	39	36	1.30	3.45	8	1	1	1	1	2	1	1
Subject 15	Bipolar Disorder	33	28	24	19	1.40	6.05	22	19	17	10	1.39	4.16	16	3	3	2	3	2	2	1
Subject 17	Bipolar Disorder	65	62	43	40	1.78	4.63	67	63	42	39	1.61	4.33	14	2	1	1	1	4	1	4
Subject 13	Bipolar Disorder	15	9	6	1	-0.54	-0.57							33	5	5	5	5	7	4	2
Subject 14	Bipolar Disorder	93	92	55	48	1.55	3.14	91	90	53	51	1.53	3.51	13	1	1	1	1	5	1	3
Subject 16	Bipolar Disorder	18	12	12	1	1.44	-0.64							29	6	4	4	2	7	5	1
Subject 18	Bipolar Disorder	33	30	26	16	1.98	4.85	63	61	37	36	1.32	3.61	12	1	2	1	2	3	2	1
Subject 19	Bipolar Disorder	32	30	29	15	2.29	5.75	71	69	48	24	1.79	1.83	11	1	1	1	1	3	1	3
Subject 20	Bipolar Disorder	45	43	30	27	1.42	4.28	61	60	43	39	1.81	5.00	11	1	3	1	3	1	1	1
Subject 21	Bipolar Disorder	39	36	25	14	1.33	1.95	76	75	50	49	1.72	4.67	16	3	1	2	2	2	3	3
Subject 23	Control	68	67	46	44	1.77	4.99	86	85	55	52	1.72	4.10								
Subject 24	Control	36	35	30	26	2.06	7.55	95	93	64	63	2.11	5.29								
Subject 25	Control	42	41	31	28	1.68	5.38	56	53	35	32	1.49	4.13								
Subject 26	Control	19	15	12	6	0.70	2.17	42	39	27	18	-0.61	3.78								
Subject 28	Control	33	28	23	12	1.66	2.92	27	22	19	13	1.27	5.29								
Subject 29	Control	42	40	29	20	0.62	3.97	24	21	15	8	1.07	1.80								
Subject 31	Control	28	27	25	8	1.97	2.41	62	60	39	28	1.46	2.58								
Subject 33	Control	34	33	25	23	1.52	5.25	33	30	25	9	1.24	2.28								
Subject 35	Control	23	19	13	6	-0.74	2.46	47	43	33	23	1.37	4.49								
Subject 38	Control	16	14	9	1	-0.79	-0.61	77	74	55	44	1.92	5.07								
Subject 39	Control	58	56	39	35	1.64	4.45	97	96	58	57	1.60	3.96								
Subject 40	Control	42	39	34	19	2.17	4.66	58	56	43	37	2.17	5.88								
Subject 22	Control	70	68	49	47	1.62	6.01	88	86	60	59	2.12	5.55								
Subject 27	Control	39	37	31	21	2.00	5.57	100	99	54	54	1.26	3.44								
Subject 30	Control	45	43	36	28	2.17	6.46	76	75	51	50	1.84	4.76								
Subject 32	Control	41	39	31	23	1.78	5.11	52	51	39	35	1.99	6.01								
Subject 34	Control	34	30	26	11	1.90	2.91	28	23	11	1	-2.17	-0.69								
Subject 36	Control	24	21	15	9	-0.41	3.67	26	20	10	1	-1.64	-0.61								
Subject 37	Control	36	35	30	27	2.06	7.72	81	80	48	47	1.41	3.65								
Subject 41	Control	31	29	26	9	1.91	2.30	57	55	39	36	1.81	4.98								
Subject 42	Control	33	31	25	14	1.60	3.42	61	59	39	39	1.64	4.46								
