# Supplementary material for: Thought disorder measured as random speech structure classifies negative symptoms and schizophrenia diagnosis 6 months in advance
Source: NPJ Schizophr. 2017 Apr 13;3:18. doi: 10.1038/s41537-017-0019-3 (PMC5441540; doi:10.1038/s41537-017-0019-3)
Supplement: Supplementary file 9 — Supplementary Table 9 [file 41537_2017_19_MOESM9_ESM.pdf]

Supplementary Table 9: Demographic data and indices from recent-onset psychosis sample.

		Demographic/Medication				Dirsorganization Index		
**NoID Subjects**	**Group**	**APDose**	**Age**	**Education**	**Sex**	**Dream+Negative**	**Dream**	**Negative**
Subject 01	Schizophrenia	414	16	8	m	24.13	20.68	25.17
Subject 02	Schizophrenia	157	18	9	m	18.32	25.87	15.12
Subject 04	Schizophrenia	132	18	6	m	14.36	16.29	17.71
Subject 05	Schizophrenia	7	9	3	m	23.41	21.78	20.64
Subject 07	Schizophrenia	91	15	6	m			31.23
Subject 08	Schizophrenia	289	13	4	m	28.26	21.80	30.41
Subject 09	Schizophrenia	50	15	8	m	28.93	23.15	30.58
Subject 10	Schizophrenia	100	16	7	m			18.71
Subject 11	Schizophrenia	264	12	4	m			25.05
Subject 03	Schizophrenia	100	16	7	f			20.46
Subject 06	Schizophrenia	264	13	1	f	29.54	24.90	31.42
Subject 12	Bipolar Disorder	0	7	2	m	12.49	14.75	14.46
Subject 15	Bipolar Disorder	289	17	10	m	13.50	22.20	12.84
Subject 17	Bipolar Disorder	100	16	6	m	12.02	13.74	11.76
Subject 13	Bipolar Disorder	132	16	1	f			30.89
Subject 14	Bipolar Disorder	248	14	9	f	13.38	10.03	11.92
Subject 16	Bipolar Disorder	330	15	1	f			29.23
Subject 18	Bipolar Disorder	25	15	6	f	12.18	15.40	14.54
Subject 19	Bipolar Disorder	0	13	7	f	7.80	11.98	11.62
Subject 20	Bipolar Disorder	0	23	12	f	12.45	13.42	15.41
Subject 21	Bipolar Disorder	66	17	10	f	16.25	11.02	20.82
Subject 23	Control		14	9	m	8.61	9.37	10.42
Subject 24	Control		19	11	m	0.51	6.32	8.39
Subject 25	Control		16	9	m	11.52	16.09	12.85
Subject 26	Control		8	2	m	20.30	18.86	23.65
Subject 28	Control		13	6	m	20.28	21.51	19.22
Subject 29	Control		8	2	m	18.77	22.87	15.82
Subject 31	Control		14	9	m	17.33	14.85	19.37
Subject 33	Control		15	6	m	13.80	19.62	14.56
Subject 35	Control		17	7	m	18.42	16.85	22.75
Subject 38	Control		16	7	m	21.02	9.47	30.08
Subject 39	Control		21	12	m	9.10	8.33	12.95
Subject 40	Control		15	9	m	11.43	13.44	12.75
Subject 22	Control		18	11	f	5.25	7.66	7.58
Subject 27	Control		13	7	f	7.09	9.67	11.88
Subject 30	Control		23	12	f	5.74	10.69	9.05
Subject 32	Control		15	8	f	11.25	14.76	13.02
Subject 34	Control		13	7	f	21.92	24.25	18.25
Subject 36	Control		14	6	f	20.32	24.57	19.82
Subject 37	Control		19	11	f	3.40	11.70	8.12
Subject 41	Control		15	7	f	17.61	14.75	19.39
Subject 42	Control		18	11	f	15.07	14.71	17.72
