# Supplementary material for: Thought disorder measured as random speech structure classifies negative symptoms and schizophrenia diagnosis 6 months in advance
Source: NPJ Schizophr. 2017 Apr 13;3:18. doi: 10.1038/s41537-017-0019-3 (PMC5441540; doi:10.1038/s41537-017-0019-3)
Supplement: Supplementary file 10 — Supplementary Table 10 [file 41537_2017_19_MOESM10_ESM.pdf]

Supplementary Table 10: Raw data from an independent cohort of chronic psychotic sample (20 patients with schizophrenia diagnosis, 20 patients with bipolar disorder diagnosis and 20 matched control) (initials, diagnostic group, connectedness graph attributes from dream reports - average of 30-words graphs, comprising edges (E), largest connected component (LCC)).

		Dream			Dirsorganization Index	PANSS negative
**NoID Subjects**	**Diagnostic Group**	**Edges**	**LCC**	**LSC**	**Dream**	**Total**
Subject 01	Schizophrenia	21.00	16.00	1.00	29.49	27
Subject 02	Schizophrenia	25.96	17.38	8.98	15.88	20
Subject 04	Schizophrenia	27.39	23.71	14.47	12.61	13
Subject 05	Schizophrenia	25.81	15.90	8.79	16.30	17
Subject 07	Schizophrenia	27.82	21.06	11.92	10.76	16
Subject 08	Schizophrenia	24.45	19.86	8.30	20.37	29
Subject 09	Schizophrenia	25.92	20.08	10.55	16.31	16
Subject 10	Schizophrenia	28.51	24.52	17.82	9.85	16
Subject 01	Schizophrenia	24.58	17.01	6.62	19.64	9
Subject 02	Schizophrenia	18.97	12.70	1.00	35.73	33
Subject 04	Schizophrenia	27.25	21.07	9.49	12.00	9
Subject 05	Schizophrenia	28.58	23.49	17.13	9.48	8
Subject 07	Schizophrenia	25.05	15.76	5.49	17.94	11
Subject 08	Schizophrenia	24.44	17.65	4.47	19.63	26
Subject 09	Schizophrenia	25.83	21.71	8.23	16.12	20
Subject 10	Schizophrenia	25.40	20.40	6.40	17.06	37
Subject 01	Schizophrenia	25.76	19.69	16.24	17.99	27
Subject 02	Schizophrenia	25.84	18.90	9.10	16.26	16
Subject 04	Schizophrenia	27.88	21.76	14.03	11.01	11
Subject 05	Schizophrenia	23.30	17.25	6.38	23.51	25
Subject 07	Bipolar Disorder	28.47	23.24	17.29	9.87	10
Subject 08	Bipolar Disorder	27.30	21.24	12.75	12.53	17
Subject 09	Bipolar Disorder	28.38	19.87	13.41	9.33	16
Subject 10	Bipolar Disorder	28.84	22.60	15.92	8.44	7
Subject 01	Bipolar Disorder	26.47	19.42	9.82	14.47	16
Subject 02	Bipolar Disorder	26.35	19.17	10.86	15.07	17
Subject 04	Bipolar Disorder	28.51	22.83	16.98	9.66	10
Subject 05	Bipolar Disorder	25.14	17.20	7.29	18.05	7
Subject 07	Bipolar Disorder	26.06	20.45	11.41	16.08	11
Subject 08	Bipolar Disorder	27.24	20.53	11.31	12.40	10
Subject 09	Bipolar Disorder	27.27	19.81	10.39	12.12	8
Subject 10	Bipolar Disorder	28.11	23.07	14.99	10.50	11
Subject 01	Bipolar Disorder	27.05	18.39	13.65	13.49	16
Subject 02	Bipolar Disorder	28.82	23.27	16.19	8.57	7
Subject 04	Bipolar Disorder	28.33	23.61	17.14	10.28	10
Subject 05	Bipolar Disorder	28.40	22.66	14.49	9.51	10
Subject 07	Bipolar Disorder	28.69	21.77	16.55	9.04	13
Subject 08	Bipolar Disorder	27.97	20.52	13.43	10.61	9

Subject 09	Bipolar Disorder	27.50	20.37	12.50	11.86	16
Subject 10	Bipolar Disorder	25.79	18.67	9.39	16.47	15
Subject 01	Control	28.15	23.02	15.46	10.46	7
Subject 02	Control	28.39	21.92	15.55	9.74	7
Subject 04	Control	28.06	21.48	15.70	10.78	7
Subject 05	Control	28.01	19.08	14.91	10.80	8
Subject 07	Control	28.73	23.87	19.03	9.43	10
Subject 08	Control	28.62	23.65	13.78	8.68	8
Subject 09	Control	28.65	22.87	16.44	9.15	7
Subject 10	Control	28.20	22.99	16.31	10.50	7
Subject 01	Control	28.91	22.25	18.25	8.72	13
Subject 02	Control	26.81	22.69	13.07	14.10	7
Subject 04	Control	28.77	22.54	17.50	8.99	7
Subject 05	Control	28.88	23.66	16.32	8.39	11
Subject 07	Control	28.93	25.85	16.78	8.33	8
Subject 08	Control	28.88	24.50	15.34	8.20	8
Subject 09	Control	27.85	21.70	14.75	11.26	7
Subject 10	Control	27.73	24.11	14.29	11.52	7
Subject 01	Control	29.00	25.28	18.29	8.44	7
Subject 02	Control	28.22	24.32	13.95	9.95	7
Subject 04	Control	26.27	20.05	13.17	15.80	16
Subject 05	Control	28.30	22.62	17.92	10.52	9
