# Supplementary material for: Thought disorder measured as random speech structure classifies negative symptoms and schizophrenia diagnosis 6 months in advance
Source: NPJ Schizophr. 2017 Apr 13;3:18. doi: 10.1038/s41537-017-0019-3 (PMC5441540; doi:10.1038/s41537-017-0019-3)
Supplement: Supplementary file 11 — Supplementary Information [file 41537_2017_19_MOESM11_ESM.docx]

**Supplementary Information (SI):**

Composed by 10 tables - statistical information (classification using different reports, comparison of connectedness, correlation connectedness versus PANSS, correlation connectedness versus confound factors, comparison of Disorganization Indexes, adjusted correlations for confound factors and validation of Disorganization Index across samples), 3 tables with Raw Data (from recent-onset psychosis and chronic-psychosis sample).

**Supplementary Table 1:** Classification quality to classify Schizophrenia group from others subjects using a Naïve Bayes classifier with all 5 connectedness attributes (E, LCC, LSC, LCCz, LSCz) from different time-limited memory reports.

| **Groups** | **Sensitivity** | **Specificity** | **Precision** | **Recall** | **F-Measure** | **AUC** | **Accuracy** |
| --- | --- | --- | --- | --- | --- | --- | --- |
| **Dream** | 0.81 | 0.85 | 0.87 | 0.81 | 0.82 | 0.84 | 80.56 |
| **Negative** | 0.76 | 0.68 | 0.78 | 0.76 | 0.77 | 0.78 | 76.19 |
| **Positive** | 0.69 | 0.77 | 0.79 | 0.69 | 0.71 | 0.74 | 69.05 |
| **Neutral** | 0.69 | 0.71 | 0.76 | 0.69 | 0.71 | 0.63 | 69.05 |
| **Yesterday** | 0.69 | 0.54 | 0.70 | 0.69 | 0.70 | 0.64 | 69.05 |
| **Oldest** | 0.57 | 0.56 | 0.66 | 0.57 | 0.60 | 0.62 | 57.14 |

**Supplementary Table 2:** Statistical comparison of connectedness attributes (E, LCC, LSC, LCCz, LSCz) between diagnostic groups (Schizophrenia = S, Bipolar = B, Control = C). KS test rejects normal distribution of all samples (Bonferroni corrected for 2 comparisons, p < 0.0250 in bold), Levene’s test verifies variance homogeneity (Bonferroni corrected for 2 comparisons, p < 0.0250 in bold). Kruskal-Wallis test (SxBxC, Bonferroni corrected for 2 comparisons (2 memory reports), p < 0.0250 in bold); Wilcoxon-Ranksum test (SxB, SxC, SxB+C, BxC; Bonferroni corrected for 6 comparisons (4 comparison for each memory reports), p < 0.0063 in bold). Statistical comparison using Wilcoxon-Ranksum test between male x female in Control group showed no difference (Bonferroni corrected for 2 comparisons (2 memory reports), p < 0.0250 in bold)).

| **KS test** | | **E** | **LCC** | **LSC** | **LCCz** | **LSCz** |
| --- | --- | --- | --- | --- | --- | --- |
| **Dream** | p value | **3.60E-33** | **3.60E-33** | **5.45E-25** | **4.59E-16** | **1.01E-22** |
|  | h | 1 | 1 | 1 | 1 | 1 |
| **Negative** | p value | **2.39E-38** | **9.68E-37** | **2.42E-27** | **2.66E-16** | **4.70E-21** |
|  | h | 1 | 1 | 1 | 1 | 1 |
| **Levene’s Test** | | **E** | **LCC** | **LSC** | **LCCz** | **LSCz** |
| **Dream** | | 0.0804 | 0.1266 | 0.0349 | 0.4017 | 0.0603 |
| **Negative** | | 0.1264 | 0.3544 | 0.095 | 0.8135 | 0.7008 |
| **Kruskal-Wallis** | | **E** | **LCC** | **LSC** | **LCCz** | **LSCz** |
| **Dream** | S x B x C | **0.0070** | **0.0074** | **0.0112** | 0.3976 | 0.2240 |
| **Negative** | S x B x C | **0.0021** | **0.0056** | **0.0034** | 0.3197 | **0.0158** |
| **Wilcoxon Ranksum** | | **E** | **LCC** | **LSC** | **LCCz** | **LSCz** |
| **Dream** | SxB | **0.0056** | **0.0031** | **0.0040** | 0.1893 | 0.1893 |
|  | SxC | **0.0042** | **0.0045** | 0.0079 | 0.2652 | 0.1239 |
|  | Sx(B+C) | **0.0021** | **0.0019** | **0.0031** | 0.1872 | 0.1013 |
|  | BxC | 0.5418 | 0.8640 | 0.8642 | 0.9029 | 0.5419 |
| **Negative** | SxB | 0.0081 | 0.0181 | 0.0205 | 0.3418 | 0.1300 |
|  | SxC | **0.0009** | **0.0022** | **0.0009** | 0.1421 | **0.0033** |
|  | Sx(B+C) | **0.0005** | **0.0015** | **0.0008** | 0.1446 | **0.0060** |
|  | BxC | 0.7997 | 0.6719 | 0.8823 | 0.7513 | 0.4856 |
| **Wilcoxon Ranksum** | | **E** | **LCC** | **LSC** | **LCCz** | **LSCz** |
| **Dream** | Male x Fem | 0.6959 | 1.0000 | 0.6440 | 0.9717 | 0.9151 |
| **Negative** | Male x Fem | 0.6439 | 0.3922 | 0.5936 | 0.2707 | 0.2707 |

**Supplementary Table 3:** Spearman correlation between connectedness attributes (E, LCC, LSC, LCCz, LSCz) and negative symptoms measured by PANSS (total negative subscale, N1, N2, N3, N4, N5, N6, N7), using dreams or negative image reports. Showed R, and p values (significant results in bold after Bonferroni correction for 80 comparisons – 5 attributes * 2 reports * 8 symptoms, p < 0.0006).

| Dream Reports | E | | LCC | | LSC | | LCCz | | LSCz | |
| --- | --- | --- | --- | --- | --- | --- | --- | --- | --- | --- |
| PANSS Negative Subscale | Rho | p | Rho | p | Rho | p | Rho | p | Rho | p |
| Total | -0.69 | 0.0046 | -0.69 | 0.0042 | -0.65 | 0.0089 | -0.41 | 0.132 | -0.16 | 0.5654 |
| N1 | -0.71 | 0.0028 | -0.71 | 0.0031 | -0.72 | 0.0026 | -0.34 | 0.2121 | -0.23 | 0.4098 |
| N2 | -0.85 | **0.0001** | -0.8 | **0.0003** | -0.76 | 0.0009 | -0.39 | 0.1463 | -0.2 | 0.4775 |
| N3 | -0.57 | 0.0279 | -0.57 | 0.0279 | -0.56 | 0.0286 | -0.25 | 0.3755 | -0.11 | 0.6962 |
| N4 | -0.56 | 0.0317 | -0.48 | 0.0724 | -0.4 | 0.1392 | -0.11 | 0.6852 | 0.33 | 0.2355 |
| N5 | -0.44 | 0.0978 | -0.49 | 0.0634 | -0.47 | 0.0757 | -0.39 | 0.1459 | -0.46 | 0.0836 |
| N6 | -0.6 | 0.0192 | -0.6 | 0.0183 | -0.57 | 0.0281 | -0.44 | 0.0988 | -0.2 | 0.4774 |
| N7 | 0.63 | 0.0126 | 0.64 | 0.0101 | 0.6 | 0.0184 | 0.28 | 0.3200 | 0.33 | 0.2342 |
| Negative Image Reports | E | | LCC | | LSC | | LCCz | | LSCz | |
| PANSS Negative Subscale | Rho | p | Rho | p | Rho | p | Rho | p | Rho | p |
| Total | -0.81 | **0.0000** | -0.85 | **0.0000** | -0.81 | **0.0000** | -0.7 | **0.0005** | -0.77 | **0.0001** |
| N1 | -0.78 | **0.0000** | -0.8 | **0.0000** | -0.77 | **0.0000** | -0.63 | 0.0021 | -0.69 | **0.0006** |
| N2 | -0.77 | **0.0000** | -0.77 | **0.0001** | -0.75 | **0.0001** | -0.62 | 0.0027 | -0.67 | 0.0008 |
| N3 | -0.8 | **0.0000** | -0.77 | **0.0000** | -0.82 | **0.0000** | -0.59 | 0.0051 | -0.75 | **0.0001** |
| N4 | -0.69 | **0.0006** | -0.73 | **0.0002** | -0.62 | 0.0026 | -0.69 | **0.0005** | -0.57 | 0.0065 |
| N5 | -0.63 | 0.0024 | -0.66 | 0.0011 | -0.66 | 0.0012 | -0.46 | 0.0364 | -0.67 | 0.0008 |
| N6 | -0.8 | **0.0000** | -0.81 | **0.0000** | -0.81 | **0.0000** | -0.57 | 0.0065 | -0.73 | **0.0002** |
| N7 | 0.32 | 0.1562 | 0.26 | 0.2543 | 0.24 | 0.2998 | -0.02 | 0.9409 | 0.05 | 0.8288 |

**Supplementary Table 4:** Spearman correlations between each graph attribute and confound factors (Bonferroni corrected for 30 comparisons (2 memory reports, 3 confound factors, and 5 graph attributes, p < 0.0017).

|  | **AGE** | | | | **EDUCATION** | | | | **AP DOSE (CLPeq)** | | | |
| --- | --- | --- | --- | --- | --- | --- | --- | --- | --- | --- | --- | --- |
|  | **Dream** | | **Negative** | | **Dream** | | **Negative** | | **Dream** | | **Negative** | |
|  | **rho** | **p** | **rho** | **p** | **rho** | **p** | **rho** | **p** | **rho** | **p** | **rho** | **p** |
| **E** | -0.14 | 0.6291 | 0.17 | 0.4626 | 0.13 | 0.6455 | 0.47 | 0.0324 | -0.50 | 0.0572 | -0.42 | 0.0573 |
| **LCC** | 0.01 | 0.9746 | 0.06 | 0.7865 | 0.27 | 0.3342 | 0.40 | 0.0746 | -0.41 | 0.1270 | -0.46 | 0.0357 |
| **LSC** | -0.03 | 0.9034 | 0.35 | 0.1246 | 0.21 | 0.4563 | 0.60 | 0.0042 | -0.51 | 0.0549 | -0.30 | 0.1908 |
| **LCCz** | 0.43 | 0.1065 | -0.20 | 0.3821 | 0.32 | 0.2480 | 0.01 | 0.9529 | -0.04 | 0.8890 | -0.35 | 0.1207 |
| **LSCz** | 0.36 | 0.1879 | 0.29 | 0.1952 | 0.32 | 0.2396 | 0.50 | 0.0203 | -0.08 | 0.7798 | -0.36 | 0.1085 |

**Supplementary Table 5:** Statistical comparison of Disorganization Index between diagnostic groups (Schizophrenia = S, Bipolar = B, Control = C), considering dream + negative image reports, negative image reports or dream reports and applying the Disorganization Index from dream reports to an independent cohort of chronic psychotic sample^9^. KS test rejects normal distribution of all samples (Bonferroni corrected for 4 comparisons, p < 0.0125 in bold), Levene’s test verifies variance homogeneity (Bonferroni corrected for 4 comparisons, p < 0.0125 in bold). Kruskal-Wallis test (SxBxC, Bonferroni corrected for 3 comparisons (3 Disorganization Indexes) p < 0.0167 in bold); Wilcoxon-Ranksum test (SxB, SxC, Sx(B+C), BxC; Bonferroni corrected for 8 comparisons (4 comparison for each memory reports), p < 0.0063 in bold).

| **Disorganization Index** | | **Kruskal-Wallis (p)** | **KS test (p)** | **KS test (h)** | **Levene's test (p)** |
| --- | --- | --- | --- | --- | --- |
| **Dream + Negative** | S x B x C | **0.0035** | **2.35E-31** | 1 | 0.0472 |
| **Negative** | S x B x C | **0.0044** | **1.89E-38** | 1 | 0.6966 |
| **Dream** | S x B x C | **0.0070** | **3.60E-33** | 1 | 0.1157 |
| **Dream - Chronic Sample** | S x B x C | **8.60E-06** | **2.87E-54** | 1 | 0.0268 |
| **Disorganization Index - Wilcoxon Ranksum test (p values)** | | | | | |
| **Dream + Negative** | SxB | **0.0006** | **Negative** | SxB | 0.0221 |
|  | SxC | **0.0030** |  | SxC | **0.0013** |
|  | Sx(B+C) | **0.0009** |  | Sx(B+C) | **0.0011** |
|  | BxC | 0.7511 |  | BxC | 0.7513 |
| **Dream** | SxB | **0.0037** | **Dream - Chronic Sample** | SxB | **0.0011** |
|  | SxC | **0.0042** |  | SxC | **0.0000** |
|  | SxB+C | **0.0018** |  | SxB+C | **0.0000** |
|  | BxC | 0.8452 |  | BxC | 0.0385 |

**Supplementary Table 6:** Controls for confound factor for episode psychosis group (age, educational level, and medication status). Spearman correlation between disorganization indexes and confound factor and adjusted Spearman correlation between disorganization indexes versus negative symptoms (PANSS negative subscale), adjusted for each confound factor (Bonferroni corrected for 6 comparisons (2 memory reports and 3 confound factors, p < 0.0083).

| **Confound Factors** | **Dream+Negative** | | **Negative** | | **Dream** | |
| --- | --- | --- | --- | --- | --- | --- |
| **Disorganization Index** | **rho** | **p** | **rho** | **p** | **rho** | **p** |
| Index x Age (years) | -0.12 | 0.6688 | -0.14 | 0.5375 | -0.01 | 0.9848 |
| Index x Education (years) | -0.20 | 0.4639 | -0.42 | 0.0555 | -0.27 | 0.3380 |
| Index x AP dose (CLPeq) | 0.54 | 0.0385 | 0.32 | 0.1529 | 0.43 | 0.1108 |
| **Index x PANSS negative** | **rho** | **p** | **rho** | **p** | **rho** | **p** |
| No Adjustment | 0.92 | **0.0000** | 0.84 | **0.0000** | 0.70 | **0.0038** |
| By Age (years) | 0.92 | **0.0000** | 0.84 | **0.0000** | 0.70 | **0.0054** |
| By Education (years) | 0.91 | **0.0000** | 0.80 | **0.0000** | 0.68 | **0.0070** |
| By AP dose (CLPeq) | 0.89 | **0.0000** | 0.84 | **0.0000** | 0.61 | 0.0202 |

**Supplementary Table 7:** Validation of coefficients across different samples. Classification quality (a Naïve Bayes classifier) of sorting Schizophrenia patients from others subjects (Diagnosis), or sorting between low and high negative symptom severity (Negative Symptoms), using the Disorganization Index obtained from dream reports of the recent-onset psychotic sample (DI1), and applied to dream reports of a chronic psychotic sample ^9^ (Sample 2), or Disorganization Index obtained from dream reports of chronic psychotic sample (DI2) and applied to dream reports of the recent-onset psychotic sample (Sample 1).

|  |  | **AUC** | **Accuracy (%)** |
| --- | --- | --- | --- |
| **Sample 2 in DI1** | Diagnosis | 0.74 | 76.67 |
|  | Negative Symptoms | 0.82 | 70.00 |
| **Sample 1 in DI2** | Diagnosis | 0.81 | 80.56 |
|  | Negative Symptoms | 0.78 | 73.33 |

**Supplementary Table 8:** Raw data and PANSS from recent-onset psychosis sample.

|  | | **Negative Image** | | | | | | **Dream** | | | | | | **PANSS** | | | | | | | |
| --- | --- | --- | --- | --- | --- | --- | --- | --- | --- | --- | --- | --- | --- | --- | --- | --- | --- | --- | --- | --- | --- |
| **NoID Subjects** | **Group** | **WC** | **Edges** | **LCC** | **LSC** | **LCCz** | **LSCz** | **WC** | **Edges** | **LCC** | **LSC** | **LCCz** | **LSCz** | **Total** | **N1** | **N2** | **N3** | **N4** | **N5** | **N6** | **N7** |
| Subject 01 | Schizophrenia | 18 | 17 | 15 | 4 | 1.50 | 0.96 | 29 | 26 | 22 | 2 | 1.73 | -0.57 | 24 | 5 | 4 | 3 | 2 | 5 | 4 | 1 |
| Subject 02 | Schizophrenia | 32 | 30 | 24 | 18 | 1.68 | 4.93 | 11 | 8 | 6 | 1 | -0.09 | -0.54 | 16 | 3 | 3 | 1 | 1 | 5 | 2 | 1 |
| Subject 04 | Schizophrenia | 24 | 23 | 20 | 12 | 1.64 | 4.06 | 41 | 40 | 35 | 16 | 2.26 | 3.73 | 15 | 2 | 2 | 2 | 3 | 4 | 1 | 1 |
| Subject 05 | Schizophrenia | 31 | 29 | 26 | 7 | 1.93 | 1.63 | 32 | 31 | 18 | 17 | 0.81 | 2.80 | 21 | 3 | 3 | 3 | 2 | 6 | 3 | 1 |
| Subject 07 | Schizophrenia | 5 | 3 | 3 | 1 | 0.04 | -0.30 |  |  |  |  |  |  | 26 | 5 | 3 | 3 | 3 | 6 | 5 | 1 |
| Subject 08 | Schizophrenia | 8 | 6 | 7 | 1 | 1.70 | -0.48 | 24 | 20 | 18 | 16 | 1.07 | 8.40 | 31 | 4 | 4 | 4 | 6 | 6 | 5 | 2 |
| Subject 09 | Schizophrenia | 13 | 7 | 6 | 1 | 0.40 | -0.42 | 28 | 23 | 14 | 11 | -1.69 | 3.56 | 25 | 4 | 5 | 3 | 5 | 4 | 3 | 1 |
| Subject 10 | Schizophrenia | 30 | 27 | 26 | 8 | 1.93 | 2.58 |  |  |  |  |  |  | 20 | 5 | 4 | 4 | 1 | 1 | 4 | 1 |
| Subject 11 | Schizophrenia | 32 | 27 | 19 | 6 | 0.58 | 0.52 |  |  |  |  |  |  | 33 | 5 | 5 | 4 | 5 | 6 | 5 | 3 |
| Subject 03 | Schizophrenia | 20 | 18 | 15 | 8 | 0.77 | 3.33 |  |  |  |  |  |  | 32 | 6 | 4 | 5 | 3 | 6 | 5 | 3 |
| Subject 06 | Schizophrenia | 8 | 3 | 2 | 1 | -1.29 | -0.25 | 14 | 9 | 9 | 1 | 1.33 | -0.64 | 34 | 6 | 5 | 5 | 4 | 7 | 6 | 1 |
| Subject 12 | Bipolar Disorder | 34 | 31 | 28 | 14 | 2.10 | 4.53 | 67 | 63 | 39 | 36 | 1.30 | 3.45 | 8 | 1 | 1 | 1 | 1 | 2 | 1 | 1 |
| Subject 15 | Bipolar Disorder | 33 | 28 | 24 | 19 | 1.40 | 6.05 | 22 | 19 | 17 | 10 | 1.39 | 4.16 | 16 | 3 | 3 | 2 | 3 | 2 | 2 | 1 |
| Subject 17 | Bipolar Disorder | 65 | 62 | 43 | 40 | 1.78 | 4.63 | 67 | 63 | 42 | 39 | 1.61 | 4.33 | 14 | 2 | 1 | 1 | 1 | 4 | 1 | 4 |
| Subject 13 | Bipolar Disorder | 15 | 9 | 6 | 1 | -0.54 | -0.57 |  |  |  |  |  |  | 33 | 5 | 5 | 5 | 5 | 7 | 4 | 2 |
| Subject 14 | Bipolar Disorder | 93 | 92 | 55 | 48 | 1.55 | 3.14 | 91 | 90 | 53 | 51 | 1.53 | 3.51 | 13 | 1 | 1 | 1 | 1 | 5 | 1 | 3 |
| Subject 16 | Bipolar Disorder | 18 | 12 | 12 | 1 | 1.44 | -0.64 |  |  |  |  |  |  | 29 | 6 | 4 | 4 | 2 | 7 | 5 | 1 |
| Subject 18 | Bipolar Disorder | 33 | 30 | 26 | 16 | 1.98 | 4.85 | 63 | 61 | 37 | 36 | 1.32 | 3.61 | 12 | 1 | 2 | 1 | 2 | 3 | 2 | 1 |
| Subject 19 | Bipolar Disorder | 32 | 30 | 29 | 15 | 2.29 | 5.75 | 71 | 69 | 48 | 24 | 1.79 | 1.83 | 11 | 1 | 1 | 1 | 1 | 3 | 1 | 3 |
| Subject 20 | Bipolar Disorder | 45 | 43 | 30 | 27 | 1.42 | 4.28 | 61 | 60 | 43 | 39 | 1.81 | 5.00 | 11 | 1 | 3 | 1 | 3 | 1 | 1 | 1 |
| Subject 21 | Bipolar Disorder | 39 | 36 | 25 | 14 | 1.33 | 1.95 | 76 | 75 | 50 | 49 | 1.72 | 4.67 | 16 | 3 | 1 | 2 | 2 | 2 | 3 | 3 |
| Subject 23 | Control | 68 | 67 | 46 | 44 | 1.77 | 4.99 | 86 | 85 | 55 | 52 | 1.72 | 4.10 |  |  |  |  |  |  |  |  |
| Subject 24 | Control | 36 | 35 | 30 | 26 | 2.06 | 7.55 | 95 | 93 | 64 | 63 | 2.11 | 5.29 |  |  |  |  |  |  |  |  |
| Subject 25 | Control | 42 | 41 | 31 | 28 | 1.68 | 5.38 | 56 | 53 | 35 | 32 | 1.49 | 4.13 |  |  |  |  |  |  |  |  |
| Subject 26 | Control | 19 | 15 | 12 | 6 | 0.70 | 2.17 | 42 | 39 | 27 | 18 | -0.61 | 3.78 |  |  |  |  |  |  |  |  |
| Subject 28 | Control | 33 | 28 | 23 | 12 | 1.66 | 2.92 | 27 | 22 | 19 | 13 | 1.27 | 5.29 |  |  |  |  |  |  |  |  |
| Subject 29 | Control | 42 | 40 | 29 | 20 | 0.62 | 3.97 | 24 | 21 | 15 | 8 | 1.07 | 1.80 |  |  |  |  |  |  |  |  |
| Subject 31 | Control | 28 | 27 | 25 | 8 | 1.97 | 2.41 | 62 | 60 | 39 | 28 | 1.46 | 2.58 |  |  |  |  |  |  |  |  |
| Subject 33 | Control | 34 | 33 | 25 | 23 | 1.52 | 5.25 | 33 | 30 | 25 | 9 | 1.24 | 2.28 |  |  |  |  |  |  |  |  |
| Subject 35 | Control | 23 | 19 | 13 | 6 | -0.74 | 2.46 | 47 | 43 | 33 | 23 | 1.37 | 4.49 |  |  |  |  |  |  |  |  |
| Subject 38 | Control | 16 | 14 | 9 | 1 | -0.79 | -0.61 | 77 | 74 | 55 | 44 | 1.92 | 5.07 |  |  |  |  |  |  |  |  |
| Subject 39 | Control | 58 | 56 | 39 | 35 | 1.64 | 4.45 | 97 | 96 | 58 | 57 | 1.60 | 3.96 |  |  |  |  |  |  |  |  |
| Subject 40 | Control | 42 | 39 | 34 | 19 | 2.17 | 4.66 | 58 | 56 | 43 | 37 | 2.17 | 5.88 |  |  |  |  |  |  |  |  |
| Subject 22 | Control | 70 | 68 | 49 | 47 | 1.62 | 6.01 | 88 | 86 | 60 | 59 | 2.12 | 5.55 |  |  |  |  |  |  |  |  |
| Subject 27 | Control | 39 | 37 | 31 | 21 | 2.00 | 5.57 | 100 | 99 | 54 | 54 | 1.26 | 3.44 |  |  |  |  |  |  |  |  |
| Subject 30 | Control | 45 | 43 | 36 | 28 | 2.17 | 6.46 | 76 | 75 | 51 | 50 | 1.84 | 4.76 |  |  |  |  |  |  |  |  |
| Subject 32 | Control | 41 | 39 | 31 | 23 | 1.78 | 5.11 | 52 | 51 | 39 | 35 | 1.99 | 6.01 |  |  |  |  |  |  |  |  |
| Subject 34 | Control | 34 | 30 | 26 | 11 | 1.90 | 2.91 | 28 | 23 | 11 | 1 | -2.17 | -0.69 |  |  |  |  |  |  |  |  |
| Subject 36 | Control | 24 | 21 | 15 | 9 | -0.41 | 3.67 | 26 | 20 | 10 | 1 | -1.64 | -0.61 |  |  |  |  |  |  |  |  |
| Subject 37 | Control | 36 | 35 | 30 | 27 | 2.06 | 7.72 | 81 | 80 | 48 | 47 | 1.41 | 3.65 |  |  |  |  |  |  |  |  |
| Subject 41 | Control | 31 | 29 | 26 | 9 | 1.91 | 2.30 | 57 | 55 | 39 | 36 | 1.81 | 4.98 |  |  |  |  |  |  |  |  |
| Subject 42 | Control | 33 | 31 | 25 | 14 | 1.60 | 3.42 | 61 | 59 | 39 | 39 | 1.64 | 4.46 |  |  |  |  |  |  |  |  |

**Supplementary Table 9:** Demographic data and indices from recent-onset psychosis sample.

|  | | **Demographic/Medication** | | | | **Dirsorganization Index** | | |
| --- | --- | --- | --- | --- | --- | --- | --- | --- |
| **NoID Subjects** | **Group** | **APDose** | **Age** | **Education** | **Sex** | **Dream+Negative** | **Dream** | **Negative** |
| Subject 01 | Schizophrenia | 414 | 16 | 8 | m | 24.13 | 20.68 | 25.17 |
| Subject 02 | Schizophrenia | 157 | 18 | 9 | m | 18.32 | 25.87 | 15.12 |
| Subject 04 | Schizophrenia | 132 | 18 | 6 | m | 14.36 | 16.29 | 17.71 |
| Subject 05 | Schizophrenia | 7 | 9 | 3 | m | 23.41 | 21.78 | 20.64 |
| Subject 07 | Schizophrenia | 91 | 15 | 6 | m |  |  | 31.23 |
| Subject 08 | Schizophrenia | 289 | 13 | 4 | m | 28.26 | 21.80 | 30.41 |
| Subject 09 | Schizophrenia | 50 | 15 | 8 | m | 28.93 | 23.15 | 30.58 |
| Subject 10 | Schizophrenia | 100 | 16 | 7 | m |  |  | 18.71 |
| Subject 11 | Schizophrenia | 264 | 12 | 4 | m |  |  | 25.05 |
| Subject 03 | Schizophrenia | 100 | 16 | 7 | f |  |  | 20.46 |
| Subject 06 | Schizophrenia | 264 | 13 | 1 | f | 29.54 | 24.90 | 31.42 |
| Subject 12 | Bipolar Disorder | 0 | 7 | 2 | m | 12.49 | 14.75 | 14.46 |
| Subject 15 | Bipolar Disorder | 289 | 17 | 10 | m | 13.50 | 22.20 | 12.84 |
| Subject 17 | Bipolar Disorder | 100 | 16 | 6 | m | 12.02 | 13.74 | 11.76 |
| Subject 13 | Bipolar Disorder | 132 | 16 | 1 | f |  |  | 30.89 |
| Subject 14 | Bipolar Disorder | 248 | 14 | 9 | f | 13.38 | 10.03 | 11.92 |
| Subject 16 | Bipolar Disorder | 330 | 15 | 1 | f |  |  | 29.23 |
| Subject 18 | Bipolar Disorder | 25 | 15 | 6 | f | 12.18 | 15.40 | 14.54 |
| Subject 19 | Bipolar Disorder | 0 | 13 | 7 | f | 7.80 | 11.98 | 11.62 |
| Subject 20 | Bipolar Disorder | 0 | 23 | 12 | f | 12.45 | 13.42 | 15.41 |
| Subject 21 | Bipolar Disorder | 66 | 17 | 10 | f | 16.25 | 11.02 | 20.82 |
| Subject 23 | Control |  | 14 | 9 | m | 8.61 | 9.37 | 10.42 |
| Subject 24 | Control |  | 19 | 11 | m | 0.51 | 6.32 | 8.39 |
| Subject 25 | Control |  | 16 | 9 | m | 11.52 | 16.09 | 12.85 |
| Subject 26 | Control |  | 8 | 2 | m | 20.30 | 18.86 | 23.65 |
| Subject 28 | Control |  | 13 | 6 | m | 20.28 | 21.51 | 19.22 |
| Subject 29 | Control |  | 8 | 2 | m | 18.77 | 22.87 | 15.82 |
| Subject 31 | Control |  | 14 | 9 | m | 17.33 | 14.85 | 19.37 |
| Subject 33 | Control |  | 15 | 6 | m | 13.80 | 19.62 | 14.56 |
| Subject 35 | Control |  | 17 | 7 | m | 18.42 | 16.85 | 22.75 |
| Subject 38 | Control |  | 16 | 7 | m | 21.02 | 9.47 | 30.08 |
| Subject 39 | Control |  | 21 | 12 | m | 9.10 | 8.33 | 12.95 |
| Subject 40 | Control |  | 15 | 9 | m | 11.43 | 13.44 | 12.75 |
| Subject 22 | Control |  | 18 | 11 | f | 5.25 | 7.66 | 7.58 |
| Subject 27 | Control |  | 13 | 7 | f | 7.09 | 9.67 | 11.88 |
| Subject 30 | Control |  | 23 | 12 | f | 5.74 | 10.69 | 9.05 |
| Subject 32 | Control |  | 15 | 8 | f | 11.25 | 14.76 | 13.02 |
| Subject 34 | Control |  | 13 | 7 | f | 21.92 | 24.25 | 18.25 |
| Subject 36 | Control |  | 14 | 6 | f | 20.32 | 24.57 | 19.82 |
| Subject 37 | Control |  | 19 | 11 | f | 3.40 | 11.70 | 8.12 |
| Subject 41 | Control |  | 15 | 7 | f | 17.61 | 14.75 | 19.39 |
| Subject 42 | Control |  | 18 | 11 | f | 15.07 | 14.71 | 17.72 |

**Supplementary Table 10:** Raw data from an independent cohort of chronic psychotic sample (20 patients with schizophrenia diagnosis, 20 patients with bipolar disorder diagnosis and 20 matched control) (initials, diagnostic group, connectedness graph attributes from dream reports - average of 30-words graphs, comprising edges (E), largest connected component (LCC)).

|  | | **Dream** | | | **Dirsorganization Index** | **PANSS negative** |
| --- | --- | --- | --- | --- | --- | --- |
| **NoID Subjects** | **Diagnostic Group** | **Edges** | **LCC** | **LSC** | **Dream** | **Total** |
| Subject 01 | Schizophrenia | 21.00 | 16.00 | 1.00 | 29.49 | 27 |
| Subject 02 | Schizophrenia | 25.96 | 17.38 | 8.98 | 15.88 | 20 |
| Subject 04 | Schizophrenia | 27.39 | 23.71 | 14.47 | 12.61 | 13 |
| Subject 05 | Schizophrenia | 25.81 | 15.90 | 8.79 | 16.30 | 17 |
| Subject 07 | Schizophrenia | 27.82 | 21.06 | 11.92 | 10.76 | 16 |
| Subject 08 | Schizophrenia | 24.45 | 19.86 | 8.30 | 20.37 | 29 |
| Subject 09 | Schizophrenia | 25.92 | 20.08 | 10.55 | 16.31 | 16 |
| Subject 10 | Schizophrenia | 28.51 | 24.52 | 17.82 | 9.85 | 16 |
| Subject 01 | Schizophrenia | 24.58 | 17.01 | 6.62 | 19.64 | 9 |
| Subject 02 | Schizophrenia | 18.97 | 12.70 | 1.00 | 35.73 | 33 |
| Subject 04 | Schizophrenia | 27.25 | 21.07 | 9.49 | 12.00 | 9 |
| Subject 05 | Schizophrenia | 28.58 | 23.49 | 17.13 | 9.48 | 8 |
| Subject 07 | Schizophrenia | 25.05 | 15.76 | 5.49 | 17.94 | 11 |
| Subject 08 | Schizophrenia | 24.44 | 17.65 | 4.47 | 19.63 | 26 |
| Subject 09 | Schizophrenia | 25.83 | 21.71 | 8.23 | 16.12 | 20 |
| Subject 10 | Schizophrenia | 25.40 | 20.40 | 6.40 | 17.06 | 37 |
| Subject 01 | Schizophrenia | 25.76 | 19.69 | 16.24 | 17.99 | 27 |
| Subject 02 | Schizophrenia | 25.84 | 18.90 | 9.10 | 16.26 | 16 |
| Subject 04 | Schizophrenia | 27.88 | 21.76 | 14.03 | 11.01 | 11 |
| Subject 05 | Schizophrenia | 23.30 | 17.25 | 6.38 | 23.51 | 25 |
| Subject 07 | Bipolar Disorder | 28.47 | 23.24 | 17.29 | 9.87 | 10 |
| Subject 08 | Bipolar Disorder | 27.30 | 21.24 | 12.75 | 12.53 | 17 |
| Subject 09 | Bipolar Disorder | 28.38 | 19.87 | 13.41 | 9.33 | 16 |
| Subject 10 | Bipolar Disorder | 28.84 | 22.60 | 15.92 | 8.44 | 7 |
| Subject 01 | Bipolar Disorder | 26.47 | 19.42 | 9.82 | 14.47 | 16 |
| Subject 02 | Bipolar Disorder | 26.35 | 19.17 | 10.86 | 15.07 | 17 |
| Subject 04 | Bipolar Disorder | 28.51 | 22.83 | 16.98 | 9.66 | 10 |
| Subject 05 | Bipolar Disorder | 25.14 | 17.20 | 7.29 | 18.05 | 7 |
| Subject 07 | Bipolar Disorder | 26.06 | 20.45 | 11.41 | 16.08 | 11 |
| Subject 08 | Bipolar Disorder | 27.24 | 20.53 | 11.31 | 12.40 | 10 |
| Subject 09 | Bipolar Disorder | 27.27 | 19.81 | 10.39 | 12.12 | 8 |
| Subject 10 | Bipolar Disorder | 28.11 | 23.07 | 14.99 | 10.50 | 11 |
| Subject 01 | Bipolar Disorder | 27.05 | 18.39 | 13.65 | 13.49 | 16 |
| Subject 02 | Bipolar Disorder | 28.82 | 23.27 | 16.19 | 8.57 | 7 |
| Subject 04 | Bipolar Disorder | 28.33 | 23.61 | 17.14 | 10.28 | 10 |
| Subject 05 | Bipolar Disorder | 28.40 | 22.66 | 14.49 | 9.51 | 10 |
| Subject 07 | Bipolar Disorder | 28.69 | 21.77 | 16.55 | 9.04 | 13 |
| Subject 08 | Bipolar Disorder | 27.97 | 20.52 | 13.43 | 10.61 | 9 |
| Subject 09 | Bipolar Disorder | 27.50 | 20.37 | 12.50 | 11.86 | 16 |
| Subject 10 | Bipolar Disorder | 25.79 | 18.67 | 9.39 | 16.47 | 15 |
| Subject 01 | Control | 28.15 | 23.02 | 15.46 | 10.46 | 7 |
| Subject 02 | Control | 28.39 | 21.92 | 15.55 | 9.74 | 7 |
| Subject 04 | Control | 28.06 | 21.48 | 15.70 | 10.78 | 7 |
| Subject 05 | Control | 28.01 | 19.08 | 14.91 | 10.80 | 8 |
| Subject 07 | Control | 28.73 | 23.87 | 19.03 | 9.43 | 10 |
| Subject 08 | Control | 28.62 | 23.65 | 13.78 | 8.68 | 8 |
| Subject 09 | Control | 28.65 | 22.87 | 16.44 | 9.15 | 7 |
| Subject 10 | Control | 28.20 | 22.99 | 16.31 | 10.50 | 7 |
| Subject 01 | Control | 28.91 | 22.25 | 18.25 | 8.72 | 13 |
| Subject 02 | Control | 26.81 | 22.69 | 13.07 | 14.10 | 7 |
| Subject 04 | Control | 28.77 | 22.54 | 17.50 | 8.99 | 7 |
| Subject 05 | Control | 28.88 | 23.66 | 16.32 | 8.39 | 11 |
| Subject 07 | Control | 28.93 | 25.85 | 16.78 | 8.33 | 8 |
| Subject 08 | Control | 28.88 | 24.50 | 15.34 | 8.20 | 8 |
| Subject 09 | Control | 27.85 | 21.70 | 14.75 | 11.26 | 7 |
| Subject 10 | Control | 27.73 | 24.11 | 14.29 | 11.52 | 7 |
| Subject 01 | Control | 29.00 | 25.28 | 18.29 | 8.44 | 7 |
| Subject 02 | Control | 28.22 | 24.32 | 13.95 | 9.95 | 7 |
| Subject 04 | Control | 26.27 | 20.05 | 13.17 | 15.80 | 16 |
| Subject 05 | Control | 28.30 | 22.62 | 17.92 | 10.52 | 9 |
